# Ultrasensitive Ion‐Imprinted Detection System with Pore‐Depended Electrochemiluminescence Mechanism for Accurate and Rapid Monitoring of Cesium in the Environment

**DOI:** 10.1002/advs.202516113

**Published:** 2025-10-30

**Authors:** Ziyu Wang, Lei Fang, Jianing Zhao, Chengqi Li, Hebing Xie, Jian‐Bin Pan, Daoben Hua

**Affiliations:** ^1^ State Key Laboratory of Radiation Medicine and Protection School for Radiological and Interdisciplinary Sciences (RAD‐X) Collaborative Innovation Center of Radiological Medicine of Jiangsu Higher Education Institutions Soochow University 199 Ren'ai Road Suzhou 215123 China; ^2^ State Key Laboratory of Analytical Chemistry for Life Science School of Chemistry and Chemical Engineering Nanjing University Nanjing 210023 China; ^3^ Tibet God Monkey Pharmaceutical Co., Ltd Shigatse 858300 China

**Keywords:** cesium, electrochemiluminescence, environmental determination, sensor, trace detection

## Abstract

There is growing global concern that cesium‐137 poses a potential risk to the environment, ecology, and public health. For the first time, an ultrasensitive cesium detection system with a pore‐dependent electrochemiluminescence mechanism is developed in this work for the accurate and rapid monitoring in the environment. A cesium‐imprinted film is prepared on the electrode to obtain an electrochemiluminescence sensor with cesium‐matched pores. Tri‐*n*‐propylamine (TPrA) can enter the cesium‐matched pores and give an electrochemical oxidation process, while Ru(bpy)_3_^2+^ cannot. When cesium ions can selectively bind to the ─N═ group to occupy the pores, they block the oxidation process of TPrA in pores to quench the electrochemiluminescence signal of Ru(bpy)_3_^2+^ with an ultralow limit of detection (50 pg L^−1^). It is successfully employed to the environmental sample (salt water, fresh water, and different animals) determination and the cesium accumulation monitoring in aquatic animals, indicating its application in environmental and ecology research. A chip‐type detection system is designed basing on this sensor to realize real‐time detection. This work not only aids in environmental monitoring efforts but also contributes to the broader scientific understanding of cesium mobility behavior in aquatic environments, making it important for the fields of environment, ecology, and public health.

## Graphical abstract

This study focuses on the rapid and accurate determination of cesium, a key radioactive contaminant attracting global attention, by creating an ultrasensitive pore‐dependent electrochemiluminescence detection system. It has been successfully applied in the determination of various kinds of environmental samples, exhibiting its significance in the fields of environment, ecology, and public health.

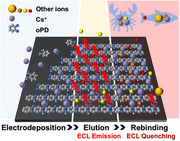

## 1 Introduction

The challenge posed by radioactive contaminants is currently attracting significant global attention.^[^ ^1^, ^2^, ^3^ ^]^ Among these contaminants, cesium‐137 is particularly noteworthy owing to its long half‐life of 30.2 years and considerable radiotoxicity. Cesium can participate in the metabolic process of K^+^ and tends to accumulate in soft tissue, inducing evident radiation damage.^[^ ^4^ ^]^ Radiocesium contamination in the water environment poses a significant risk to public health due to its enrichment in edible parts (such as muscle tissue) of aquatic products (fish, shrimp, crab, etc.).^[^ ^5^, ^6^ ^]^ Meanwhile, the treatment of cesium in the environment is also necessary,^[^ ^7^, ^8^, ^9^ ^]^ and the efficiency of which significantly needs to be accurately characterized. Considering the trace background of cesium in the environment (ng/L orders of magnitude), the development of methods for the accurate and rapid determination of cesium is important for both environmental monitoring and tracing of cesium contaminants.

To date, several techniques for cesium detection have been extensively studied, including methodologies such as inductively coupled plasma‒mass spectrometry (ICP‐MS),^[^ ^10^ ^]^ X‐ray fluorescence spectroscopy,^[^ ^11^ ^]^ fluorescence sensing,^[^ ^12^ ^]^ electrochemical sensing,^[^ ^13^, ^14^ ^]^ colorimetric detection,^[^ ^15^ ^]^ and radiochemistry analysis^[^ ^16^ ^]^ on. However, the limits of detection (LODs) of the known fluorescence, colorimetric, and electrochemical sensors are often on the order of nM–µM,^[^ ^14^, ^15^, ^16^, ^17^ ^]^ which cannot meet the requirements of trace cesium monitoring in the environment. Radiochemical analysis can yield ultralow LOD values (e.g., fg/L orders of magnitude) via γ‐spectrometry detection. A complex pretreatment process is often used to enrich Cs^+^ from water samples before measurement (National Eco‐Environmental Standard of P. R. China, HJ 816‐2016). ICP‐MS demonstrates good selectivity and sensitivity (LOD: ng/L orders of magnitude) in metal ion detection. However, it exhibits a poor tolerance to samples with high salinity due to the high ion interference, and the huge size of the ICP‐MS instrument also makes it unsuitable for real‐time determination in the field.

Electrochemiluminescence (ECL), which is induced by an electrochemical redox reaction on the surface of electrodes, is considered a promising choice for achieving accurate cesium monitoring in the environment because of its high sensitivity and selectivity.^[^ ^17^, ^18^ ^]^ The ultralow LOD is regarded as an important advantage of ECL technology (typically on the order of pM–aM), which can be attributed to the absence of an autoluminescent background as well as scattered light.^[^ ^19^, ^20^, ^21^ ^]^ Therefore, ECL technology has been widely applied in the development of various sensors, including sensors for cell imaging,^[^ ^19^ ^]^ chemical reaction characterization,^[^ ^22^ ^]^ and biosensors.^[^ ^23^ ^]^ Recently, ECL sensors for radioactive contaminants have also been a series of important members, which concern uranyl ion,^[^ ^24^ ^]^ iodine,^[^ ^25^ ^]^ radon^[^ ^26^ ^]^ and so on. Moreover, ECL sensors generally exhibit excellent salinity tolerance due to their high selectivity, making them particularly suitable for the analysis of high‐salinity samples such as seawater.^[^ ^27^ ^]^ The small size of the ECL instrument can be regarded as another advantage for real‐time and rapid determination. Despite these compelling attributes, to the best of our knowledge, research dedicated to the application of ECL sensors for the accurate and rapid determination of cesium is scarce.

**Figure 1:**
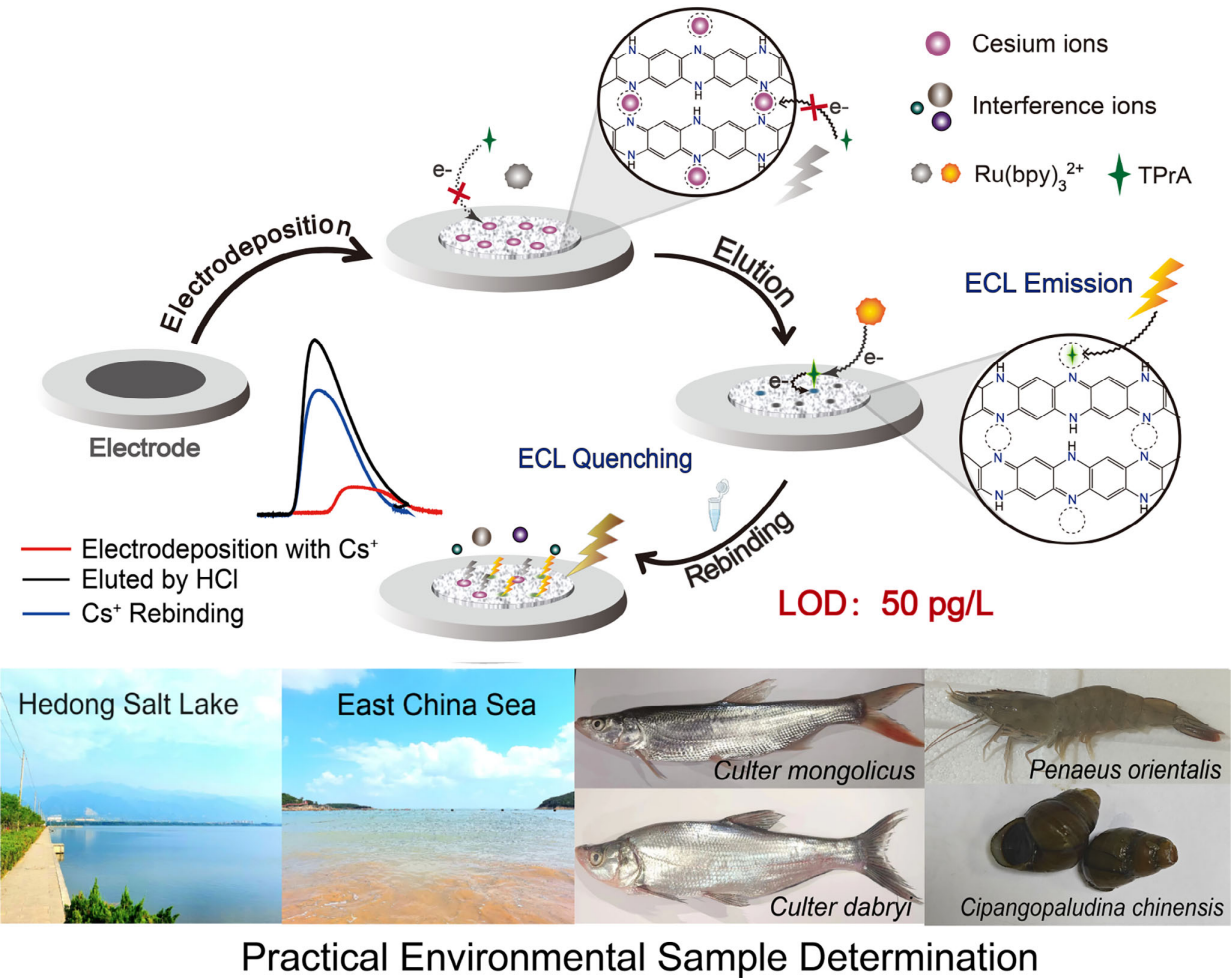
Preparation, detection mechanism, and practical applications of the cesium‐imprinted ECL sensor.

In this work, we report a cesium detection system with a pore‐dependent ECL mechanism for the accurate and rapid determination of trace cesium, which can be employed in practical environmental samples. In this study, ion‐imprinting technology was introduced to provide high selectivity for cesium ion.^[^ ^28^, ^29^, ^30^ ^]^ *O*‐phenylenediamine and cesium ions are applied to prepare a polymer film on the surface of electrodes by electrodeposition, and the bound cesium ions are then selectively eluted by HCl to provide an ECL sensor of a Cs^+^‐imprinted poly(*o*‐phenylenediamine) film (CIPF). Cs^+^ can selectively bind to the ─N═ groups to occupy the cesium‐matched pores in CIPF and block the oxidation process of tri‐*n*‐propylamine (TPrA) in these pores, resulting in a decreased ECL signal of Ru(bpy)_3_^2+^ with an ultralow LOD value of 50 pg L^−1^. Various kinds of environmental samples, including fresh water, saltwater, and different types of animal samples (fish, arthropod, and mollusk), are analyzed to demonstrate the versatility and reliability of the sensor (**Figure** **1**). The accumulation process of cesium in aquatic animal samples can also be monitored by this sensor, which confirms its application in ecology research. Moreover, a novel cesium ion ECL detection system is developed based on this sensor to realize real‐time determination. This work provides an efficient strategy for the development of an accurate and rapid detection system for trace cesium, indicating its significance for the fields of environmental monitoring, ecology, and nuclear security.

## 2 Results and Discussion

### 2.1 Synthesis and Characterization

The cesium‐binding poly(*o*‐phenylenediamine) film is fabricated using *o*‐phenylenediamine as the monomer via a modification of a procedure reported in previous literature.^[^ ^31^ ^]^ The ═N─ group in poly(*o*‐phenylenediamine) has been proven to give a good combination ability to metal ions,^[^ ^32^, ^33^ ^]^ which can be potentially employed to binding cesium. The ion‐imprinting method is further applied to realize the selective binding of Cs^+^. The film is prepared via electrodeposition with *o*‐phenylenediamine in a phosphate‐buffered saline (PBS) solution with 0.3 mM cesium ions. A PBS solution is applied to guarantee electrical conductivity to support the successful synthesis of a film combining cesium ions with poly(*o*‐phenylenediamine) (Figure S1A, Supporting Information). HCl is subsequently employed to elute the bound cesium ions to obtain the cesium‐ion selective CIPF. Higher cesium ion concentrations in the preparation of CIPF can potentially decrease sensitivity (Figure S1B, Supporting Information). This result can probably be attributed to the larger number of cesium‐matched pores, which may make the ECL signal more difficult to be quenched by trace cesium ions. Consequently, 0.3 mM cesium ion in 0.1 M PBS solution is found to be an optimal formulation for the preparation of CIPF.

**Figure 2:**
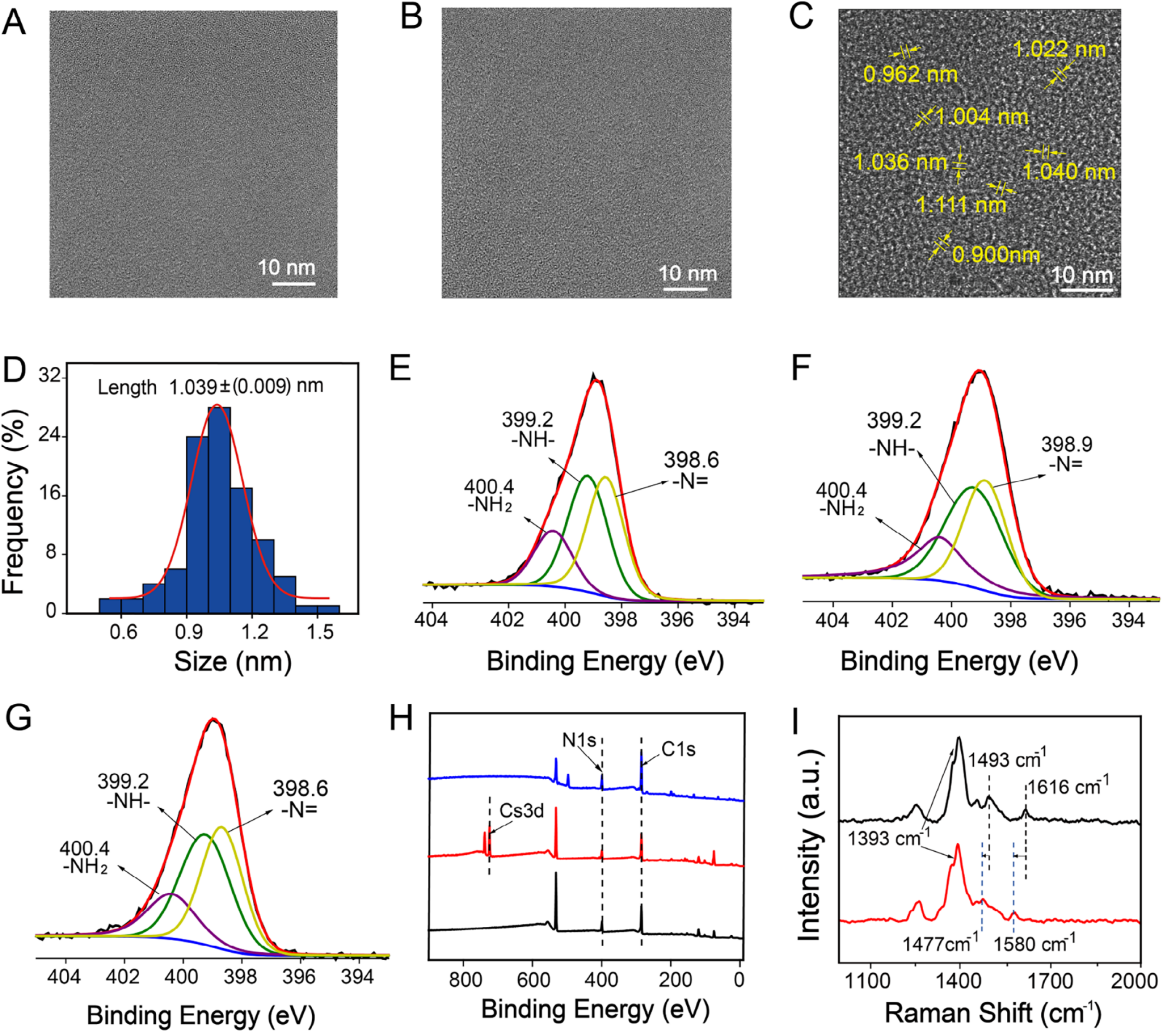
TEM and XPS of CIPF. TEM images of (A) blank poly(*o*‐phenylenediamine), B) Cs^+^‐binding poly(*o*‐phenylenediamine) film prepared with 0.3 mM Cs^+^, and C) CIPF. D) The distribution of pore diameter values in Figure 2C. N1s XPS data of (E) blank poly(o‐phenylenediamine), F) Cs^+^‐binding poly(*o*‐phenylenediamine) film prepared with 0.3 mM Cs^+^, and G) CIPF. H) The whole XPS data of blank poly(*o*‐phenylenediamine) (blue), Cs^+^‐binding poly(o‐phenylenediamine) film prepared with 0.3 mM Cs^+^ (red), and CIPF (black). I) Raman spectra of Cs^+^‐binding poly(*o*‐phenylenediamine) film prepared with (red) and without (black) 0.3 mM Cs^+^.

According to the high‐resolution transmission electron microscope (TEM) images, both poly(*o*‐phenylenediamine) films with and without Cs^+^ combination have a relatively planar surface (**Figure** **2**A,B), while the surface becomes clearly porous after the cesium ions are eluted by HCl (CIPF, Figure 2C). The diameter values of most pores are distributed in the range of 0.9–1.2 nm, with the average value calculated as ≈1.04 nm (Figure 2D) via the method in Supporting Information. Based on the relevant data provided in previous studies, the hydrated diameter of cesium ions can be calculated as 1 nm,^[^ ^34^ ^]^ which corresponds well with the dimensions of the pores. The methodologies for the analysis of pore size and the calculation of hydrated diameter values are described in the Supporting Information. This result confirms that the Cs^+^‐imprinted pores produced by HCl elution can match cesium ions well, indicating the potential selectivity of CIPF for cesium ions.

According to the X‐ray photoelectron spectroscopy (XPS) data, the peaks of the ─N═ and ─NH─ groups of the blank poly(*o*‐phenylenediamine) film exhibit similar integrated areas (Figure 2E), demonstrating the chemical structure of this poly(*o*‐phenylenediamine) film (Figure 1). After the binding of cesium ions, only the peak of ─N═ shifted from 398.6 eV to 398.9 eV for the film combining cesium ions with the poly(*o*‐phenylenediamine) (Figure 2F). After the elution of Cs^+^, this peak returned 398.6 eV in CIPF (Figure 2G), confirming that the Cs^+^‐binding group in CIPF can be assigned to ─N═. The Cs 3d peak is observed in the film combining cesium ions with the poly(*o*‐phenylenediamine) and subsequently disappears after elution by HCl (CIPF), confirming the elution of cesium ions (Figure 2H). Raman spectroscopy is also conducted to investigate Cs^+^‐binding effects on the poly(*o*‐phenylenediamine) framework (Figure 2I). Compared with blank poly(*o*‐phenylenediamine) film (black), the C═N stretching vibration shifts from 1616 cm^−1^ to 1580 cm^−1^ while the C═N coupling vibration shifts from 1493 cm^−1^ to 1477 cm^−1^ after the incorporation of Cs^+^ (blue), indicating the combination of ═N─ group with Cs^+^. The transient intensity variations in C─N⁺• (≈1370 cm^−1^) and C─N (≈1250 cm^−1^) modes reflect localized electronic perturbations. This result confirms the Cs^+^‐driven electronic redistribution at ─N═ sites.

**Figure 3:**
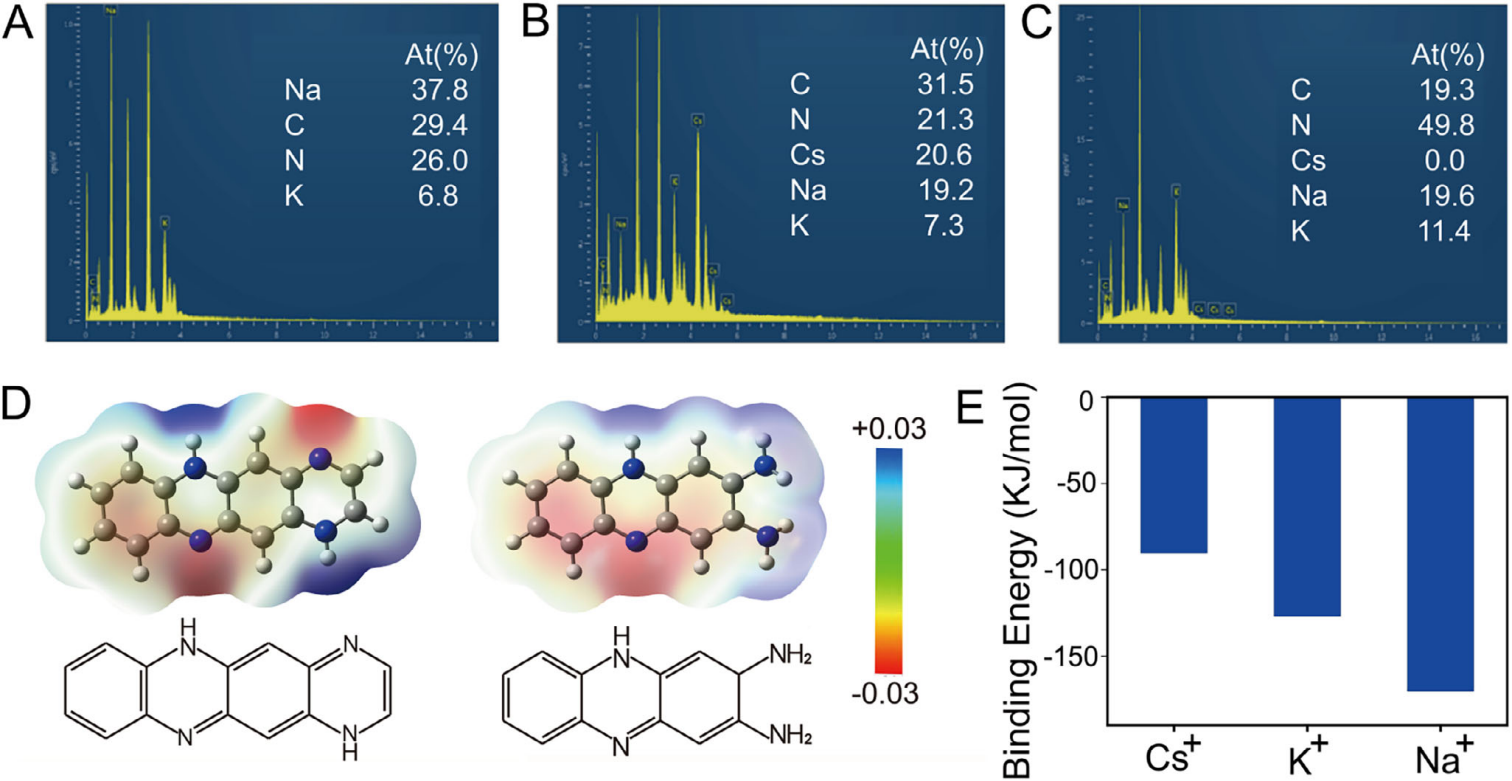
EDS data and DFT calculations. EDS data of (A) blank poly(*o*‐phenylenediamine), poly(*o*‐phenylenediamine) prepared with 40 mM Cs^+^ (B) before and (C) after elution with 1 M HCl. The DFT calculation results of (D) electron density and (E) binding energy.

The application of a PBS solution in the preparation of CIPF poses the risk of potential interference by Na^+^ and K^+^ due to their high concentration. According to the energy dispersive spectrometer (EDS) data, Cs^+^, Na^+^, and K^+^ can all bind to the poly(*o*‐phenylenediamine) film during the electrodeposition process (**Figure** **3**A,B). After elution by HCl, the Cs^+^ disappears, whereas Na^+^ and K^+^ can still be observed (Figure 3C), indicating that the imprinted Cs^+^ can be eluted by HCl, but Na^+^ and K^+^ cannot. To confirm the combination of CIPF with Cs^+^, the repeating unit (the left one in Figure 3D) and the end unit (the right one in Figure 3D) of poly(*o*‐phenylenediamine) (Figure 1) are chosen to proceed the density functional theory (DFT) calculation. As a result, the ─N═ group has the highest electron density among the polymer backbone (Figure 3D), confirming that the Cs^+^‐binding group can be assigned to the ─N═ group. The electrostatic potential energy values for the interaction of the ─N═ group with Cs^+^, Na^+^, and K^+^ are shown in Figure S2 (Supporting Information), with the corresponding results presented in Figure 3E. Among these ions, the Cs^+^ has the lowest binding energy. These results demonstrate that Cs^+^ can be eluted by HCl in the preparation of CIPF, whereas Na^+^ and K^+^ cannot, indicating that only Cs^+^‐matched pores can be obtained in the preparation of CIPF.

### 2.2 Pore‐Dependent ECL Mechanism of CIPF

**Figure 4:**
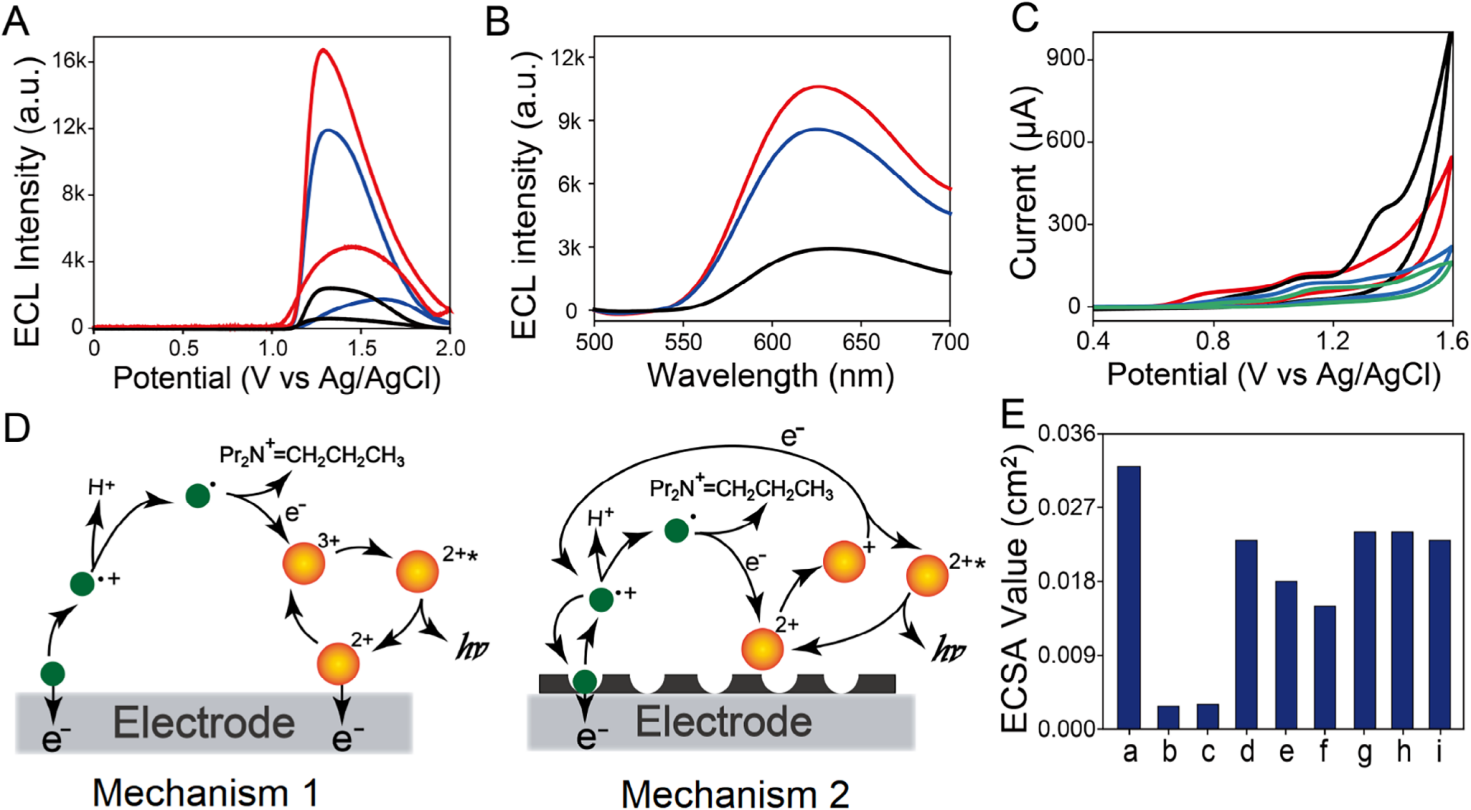
A) ECL signals and (B) ECL spectra of cesium binding poly(*o*‐phenylenediamine) (black), CIPF (red), and CIPF treated with 1 ng L^−1^ Cs^+^ solution (blue). C) CV of blank electrode (black), CIPF (red), and CIPF treated with 1 ng L^−1^ (blue)/1 µg L^−1^ (green) Cs^+^ solution in 0.1 M PBS solution (pH 7.4) with 1 mM Ru(bpy)_3_Cl_2_ and 25 mM TPrA. Scan rate: 100 mV s^−1^, PMT = 300 V. D) ECL mechanism on blank electrode (Mechanism 1) and pore‐depended ECL mechanism on CIPF modified electrode (Mechanism 2). The green is for TPrA and orange for Ru(bpy)_3_^2+^. E) ECSA values of electrochemical oxidation process of (a) blank electrode, (b) poly(*o*‐phenylenediamine), (c) cesium combined poly(*o*‐phenylenediamine), (d) CIPF, (e) CIPF treated with 10 ng L^−1^ Cs^+^, (f) CIPF treated with 10 µg L^−1^ Cs^+^, (g) CIPF treated with 40 mM Na^+^, (h) CIPF treated with 40 mM K^+^, and (i) CIPF treated with 10 µg L^−1^ Cs^+^ under 100 °C, calculated by the CV data in Figure S4D,E (Supporting Information) via the method in Supporting Information.

The ECL signals and ECL spectra of CIPF with the existence of Ru(bpy)_3_^2+^/TPrA are carried out in **Figure** **4**A,B. Compared with Cs^+^ combined poly(*o*‐phenylenediamine) film (black), CIPF exhibits a stronger ECL signal after the elution of Cs^+^ (red), which then gives an obvious quenching after being treated by 1 ng L^−1^ Cs^+^ (blue) (Figure 4A). According to Figure 4B, Cs^+^ combined poly(*o*‐phenylenediamine) film (black), CIPF (red), and Cs^+^ treated CIPF (blue) exhibit the same ECL emission peak at ≈626 nm, which is similar to that of Ru(bpy)_3_^2+^ (Figure S3, Supporting Information). This result indicates that the ECL emissions of CIPF before and after Cs^+^ treatment can be attributed to Ru(bpy)_3_^2+^. The ECL efficiency of this ECL system before and after treated by 1 ng L^−1^ cesium solution as 70% and 56% versus Ru(bpy)_3_Cl_2_ (1 mM)/TPrA (25 mM) system, respectively.

To demonstrate the ECL mechanism of this ECL device, cyclic voltammetry (CV) data have been carried out in Figure 4C. An oxidation peak can be observed in the CV of the blank electrode with Ru(bpy)_3_^2+^/TPrA (black) at ≈+1.36 V, which is similar to CV of Ru(bpy)_3_^2+^. This result indicates that this peak can be attributed to the oxidation of Ru(bpy)_3_^2+^ (Figure S4B, Supporting Information), demonstrating Mechanism 1 in Figure 4D as follows on the surface of the blank electrode.^[^ ^35^ ^]^

(1) $$\mathrm{TPrA}\;-\;e^{-}\to \mathrm{TPr}A^{\cdot +}$$

(2) $$\mathrm{TPr}A^{\cdot +}-H^{+}\to \mathrm{TPr}A^{\cdot }$$

(3) $${\mathrm{Ru}(\mathrm{bpy})}_{3}^{2+}-e^{-}\to {\mathrm{Ru}(\mathrm{bpy})}_{3}^{3+}$$

(4) $${\mathrm{Ru}(\mathrm{bpy})}_{3}^{3+}+\mathrm{TPr}A^{\cdot }\to {\mathrm{Ru}(\mathrm{bpy})}_{3}^{2+*}+Pr_{2}N^{+}=\mathrm{CHC}H_{2}CH_{3}$$

(5) $${\mathrm{Ru}(\mathrm{bpy})}_{3}^{2+*}\to {\mathrm{Ru}(\mathrm{bpy})}_{3}^{2+}+\mathrm{hv}$$

Different from the blank electrode, the CIPF‐modified electrode gives another oxidation peak at ≈+1.10 V, which is similar to the CV datum of TPrA (Figure S4A, Supporting Information). Therefore, it can be assigned to the oxidation of TPrA. Meanwhile, the oxidation peak of Ru(bpy)_3_^2+^ at ≈+1.36 V almost disappears (red, Figure 4C), which means that the oxidation process of Ru(bpy)_3_^2+^ is blocked. To further clarify this point, the diameter value of TPrA is calculated by DFT calculation as 0.79 nm (Figure S4C, Supporting Information), which is smaller than cesium‐matched pores (1.04 nm). It is known that Ru(bpy)_3_^2+^ gives the diameter value as 1.26 nm, larger than cesium‐matched pores.^[^ ^36^ ^]^ These results suggest that TPrA can enter the cesium‐matched pores to give an oxidation reaction on the surface of the electrode while Ru(bpy)_3_^2+^ cannot. We noticed the ECL process of Ru(bpy)_3_^2+^/TPrA system does not involve the oxidation of Ru(bpy)_3_^2+^ as below when the concentration of TPrA is sharply higher (1000–10 000 times) than that of Ru(bpy)_3_^2+^.^[^ ^37^, ^38^ ^]^

(6) $$\mathrm{TPrA}\;-\;e^{-}\to \;\mathrm{TPr}A^{\cdot +}$$

(7) $$\mathrm{TPr}A^{\cdot +}\;-\;H^{+}\to \;\mathrm{TPr}A^{\cdot }$$

(8) $${\mathrm{Ru}(\mathrm{bpy})}_{3}^{2+}+\mathrm{TPr}A^{\cdot }\to {\;\mathrm{Ru}(\mathrm{bpy})}_{3}^{+}+{\mathrm{Pr}}_{2}N^{+}=\mathrm{CHC}H_{2}CH_{3}$$

(9) $${\mathrm{Ru}(\mathrm{bpy})}_{3}^{+}+\mathrm{TPr}A^{\cdot +}\to {\;\mathrm{Ru}(\mathrm{bpy})}_{3}^{2+*}+\mathrm{TPrA}$$

(10) $${\mathrm{Ru}(\mathrm{bpy})}_{3}^{2+*}\to {\;\mathrm{Ru}(\mathrm{bpy})}_{3}^{2+}+\mathrm{hv}$$

In this study, considering to the smaller size (diameter: 0.79 nm) of TPrA (Figure S4C, Supporting Information) than the cesium‐matched pores, the concentration of TPrA on the surface of the electrode is obviously higher than that of Ru(bpy)_3_^2+^. Therefore, the ECL process exhibits a pore‐dependent ECL mechanism on the surface of the CIPF‐modified electrode (Mechanism 2 in Figure 4D).

In order to demonstrate the mechanism, the CV data of CIPF is further measured after being treated by 10 ng L^−1^ and 10 µg L^−1^ Cs^+^ solution (blue and green, Figure 4C), respectively. It can be observed that the oxidation peak of TPrA (+1.10 V) exhibits a gradual decline as the cesium concentration increases, which can be attributed to the cesium occupation of the pores to block the access and oxidation of TPrA, thereby quenching the ECL signal.

The electrochemical surface area (ECSA) values of CIPF in electrochemical oxidation reactions are calculated by using the equation in Supporting Information and the CV data in Figure S4D,E (Supporting Information) (Figure 4E).^[^ ^39^ ^]^ The ECSA value of the blank electrode is 0.032 cm^2^, which gives a sharp decline after the modification of the cesium combined poly(*o*‐phenylenediamine) (0.003 cm^2^). After the combined Cs^+^ ions are eluted, CIPF gives a larger ECSA value as 0.023 cm^2^ due to its porous structure (Figure 2C). The ECSA values then decrease to 0.018 cm^2^ and 0.015 cm^2^ after the treatment of 10 ng L^−1^ and 10 µg L^−1^ Cs^+^, respectively, indicating the occupation of cesium‐matched pores by Cs^+^. This process decreases the active sites of electrochemical oxidation of TPrA on the surface of electrodes, which can be regarded as evidence of the Cs^+^ response mechanism of CIPF. Meanwhile, the resistance (*R*_et_) values also exhibit a similar trend (Figure S4F, Supporting Information), indicating the reliable of the calculated ECSA values. These results can also prove Mechanism 2 in Figure 4D as a pore‐dependent ECL process of CIPF, which can be applied in the determination of cesium.

### 2.3 Cesium Detection

**Figure 5:**
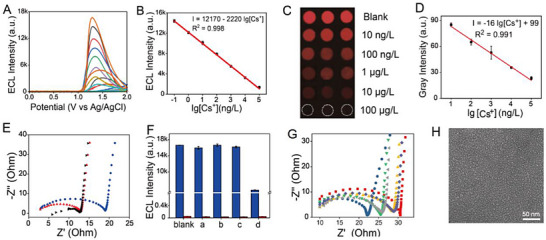
Cesium detection and selectivity mechanism. A) ECL signals of CIPF after treatment with a Cs^+^ solution at different concentrations. B) Calibration curve of the ECL intensity versus the logarithm value of the Cs^+^ concentration. C) ECL images of the blank CIPF and CIPF treated with a Cs^+^ solution at different concentrations, scan rate: 400 mV s^−1^. D) Calibration curve of the gray intensity in Figure 5C versus the logarithm value of the Cs^+^ concentration. E) Electrochemical impedance spectra of CIPF: blank (black), treated with a 1 mg L^−1^ Cs^+^ solution (blue), and treated with an interference ion solution (red). F) ECL intensity of the blank CIPF (blue)/poly(*o*‐phenylenediamine) film (red) and CIPF/poly(*o*‐phenylenediamine) film treated with (a) interference ion solution, 250 mg L^−1^ for each ion, (b) high‐salinity solution (Na^+^, K^+^, Ca^2+^, Mg^2+^, 1 g L^−1^ for each ion), (c) organic interference (acetic acid, oxalic acid, citric acid, lauryl sodium sulfate, polyethylene glycol, EDTA, glycine and ciprofloxacin hydrochloride, 250 mg L^−1^ for each one), and (d) 1 µg L^−1^ Cs^+^ solution. G) Electrochemical impedance spectra of cesium‐binding poly(*o*‐phenylenediamine) film (red), CIPF (blue), and cesium‐binding poly(*o*‐phenylenediamine) film eluted with 40 mM Na^+^ (purple)/K^+^ (orange) at room temperature and 80 °C (Na^+^: gray/K^+^: green). H) TEM image of CIPF at 100 °C. Scan rate: 100 mV s^−1^, PMT = 300 V, n = 3.

The ECL signal of CIPF can be quenched gradually after the treatment with cesium solutions of varying concentrations (**Figure** **5**A), yielding a linear range from 100 pg L^−1^ to 100 µg L^−1^ (Figure 5B) and achieving an ultralow LOD value of 50 pg L^−1^. This work provides the lowest LOD among known cesium chemical probes (Table S1, Supporting Information). Additionally, ECL imaging technology was further applied to visualize cesium (Figure 5C), and the results were similar to those shown in Figure 5A. The ECL intensity of ECL device cannot exhibit obvious changes after 14 days of storage (Figure S5, Supporting Information), indicating its long‐term stability.

Various interference ions were chosen to demonstrate the selectivity of CIPF, including Na^+^, K^+^, Ca^2+^, Mg^2+^, Sr^2+^, Mn^2+^, Cu^2+^, Zn^2+^, Pb^2+^, Ni^2+^, Fe^3+^, Al^3+^, Co^3+^, NH_4_^+^, Cl^−^, and SO_4_^2−^ with the results shown in Figure 5E,F. Among these interference ions, Na^+^, K^+^, Ca^2+^, Mg^2+^, Al^3+^, NH_4_^+^, Sr^2+^ Cl^−^, and SO_4_^2−^ are known to be common in the environment. Moreover, K^+^ is also regarded as a primary interference agent of Cs^+^ due to their similar chemical properties.^[^ ^4^ ^]^ Pb^2+^, Cu^2+^, Zn^2+^, Fe^3+^, Mn^2+^, Co^2+^, and Ni^3+^ are chosen as typical heavy metal ions. The *R*_et_ value of CIPF does not obviously change after treatment with interference ions, in contrast to the effect due to cesium ions (Figure 5E). The interference ions do not exhibit evident ECL quenching of CIPF (Figure 5F, blue). Meanwhile, some common organic interferences in the environment is also need to be focused on. Therefore, some typical organic acids, surfactants, and dissolved organic matters, including acetic acid, oxalic acid, citric acid, lauryl sodium sulfate, polyethylene glycol, ethylene diamine tetraacetic acid (EDTA), glycine, and ciprofloxacin hydrochloride, are also chosen in Figure 5F. It can be observed that these organic substances cannot affect the detection of cesium. These results indicate the high selectivity of CIPF for cesium ions. High‐salinity interference, as shown in Figure 5F, involves the addition of K^+^, Ca^2+^, Na^+^, and Mg^2+^ at a concentration of 1 g L^−1^ for each ion, and showed no obvious effect on cesium detection. This result indicates the salinity tolerance of CIPF, which is beneficial for the application of natural samples with high salinity, such as seawater. Furthermore, the prepared blank poly(*o*‐phenylenediamine) film does not give an evident response to both interference ions and the cesium concentration (Figure 5F, red). The non‐pore structure causes the decline of the ECSA of the poly(*o*‐phenylenediamine) film (0.003 cm^2^, Figure 4E,b) compared with CIPF (0.023 cm^2^, Figure 4E,d), which blocks the ECL process. The unchanged *R*_et_ value of the blank poly(*o*‐phenylenediamine) film after elution with HCl (Figure S6, Supporting Information) can also prove this result. On the other hand, the enlarged ECL signals of poly(*o*‐phenylenediamine) film in Figure 5F and Figure S7 (Supporting Information) indicate no obvious selectivity to cesium under high interference with the absence of cesium‐matched pores. Due to the stronger binding of Na^+^ and K^+^ with the ─N═ group compared to Cs^+^ (Figure 3E), the selectivity mechanism of this sensor under the interference of Na^+^ and K^+^ is investigated. The occupation of cesium‐matched pores by Cs^+^ in CIPF can increase the *R*_et_ value (Figure 5E). Due to the similar chemical property compared with Cs^+^,^[^ ^4^ ^]^ K^+^ is chosen as the typical interference ion to measure the selectivity coefficient *β* (K_d_(Cs^+^)/K_d_(K^+^)) value to further demonstrate the selectivity of CIPF. The calculation method is carried out in Supporting Information using the data in Figure S8 (Supporting Information). The *β* values of CIPF and poly(*o*‐phenylenediamine) film are calculated as 654.3 and 0.4, respectively. The relative selectivity coefficient K_d_’ (*β*_CIPF_/*β*_poly(o‐phenylenediamine) film_) value of CIPF versus poly(*o*‐phenylenediamine) film is 1592.5, indicating the high selectivity to Cs^+^ of CIPF after cesium imprinting.

Moreover, the hydrated diameter values of Na^+^/K^+^ (0.7/0.8 nm) are smaller than the size of the cesium‐matched pores (1.04 nm).^[^ ^31^ ^]^ Therefore, if Na^+^/K^+^ can exchange with Cs^+^, a decrease in the *R*_et_ value may be observed compared with that of cesium‐binding poly(*o*‐phenylenediamine) film because Na^+^/K^+^ cannot completely occupy the cesium‐matched pores. According to Figure 5G, the *R*_et_ value of the cesium‐binding poly(*o*‐phenylenediamine) film (red) does not obviously decrease after elution with Na^+^ (purple) and K^+^ (black) at room temperature. This result indicates that both Na^+^ and K^+^ cannot exchange with the Cs^+^ bound in the well‐matched pores.

By contrast, the *R*_et_ values decrease after elution by Na^+^ (orange) and K^+^ (green) at 80 °C, indicating that the exchange between Na^+^/K^+^ and bound Cs^+^ can proceed at higher temperatures. To clarify this phenomenon, the TEM image of CIPF at 100 °C is depicted in Figure 5H. The average size of the Cs^+^‐imprinted pores on CIPF increases from 1.04 nm at room temperature (Figure 2C) to 2.16 nm at 100 °C, suggesting that these pores are not well‐matched to Cs^+^ at higher temperatures.^[^ ^40^, ^41^ ^]^ To further demonstrate the effect of temperature to the size of Cs^+^‐imprinted pores, the TEM images of CIPF at 45, 80, and 100 °C are measured in which the average diameter value of Cs^+^‐imprinted pores gradually increases to 2.16 nm (100 °C) as the temperature rises from 25 °C (room temperature) to 100 °C (Figure S9, Supporting Information). The high cesium selectivity is attributed to the fact that the well‐matched size of the imprinted pores can selectively prevent the desorption of Cs^+^. At higher temperatures, the imprinted pores are no longer well‐matched due to their increased size, and Na^+^/K^+^ can exchange with Cs^+^ in the pores because of their higher binding energy. According to Figure 4E, the ECSA values of CIPF treated by 40 mM Na^+^, K^+^ under room temperature, as well as 10 µg L^−1^ Cs^+^ under 100 °C are 0.024, 0.024, and 0.023 cm^2^, respectively. The results are similar to that of CIPF, which can also be regarded as evidence for this mechanism.

### 2.4 Determination of Environmental Samples

**Figure 6:**
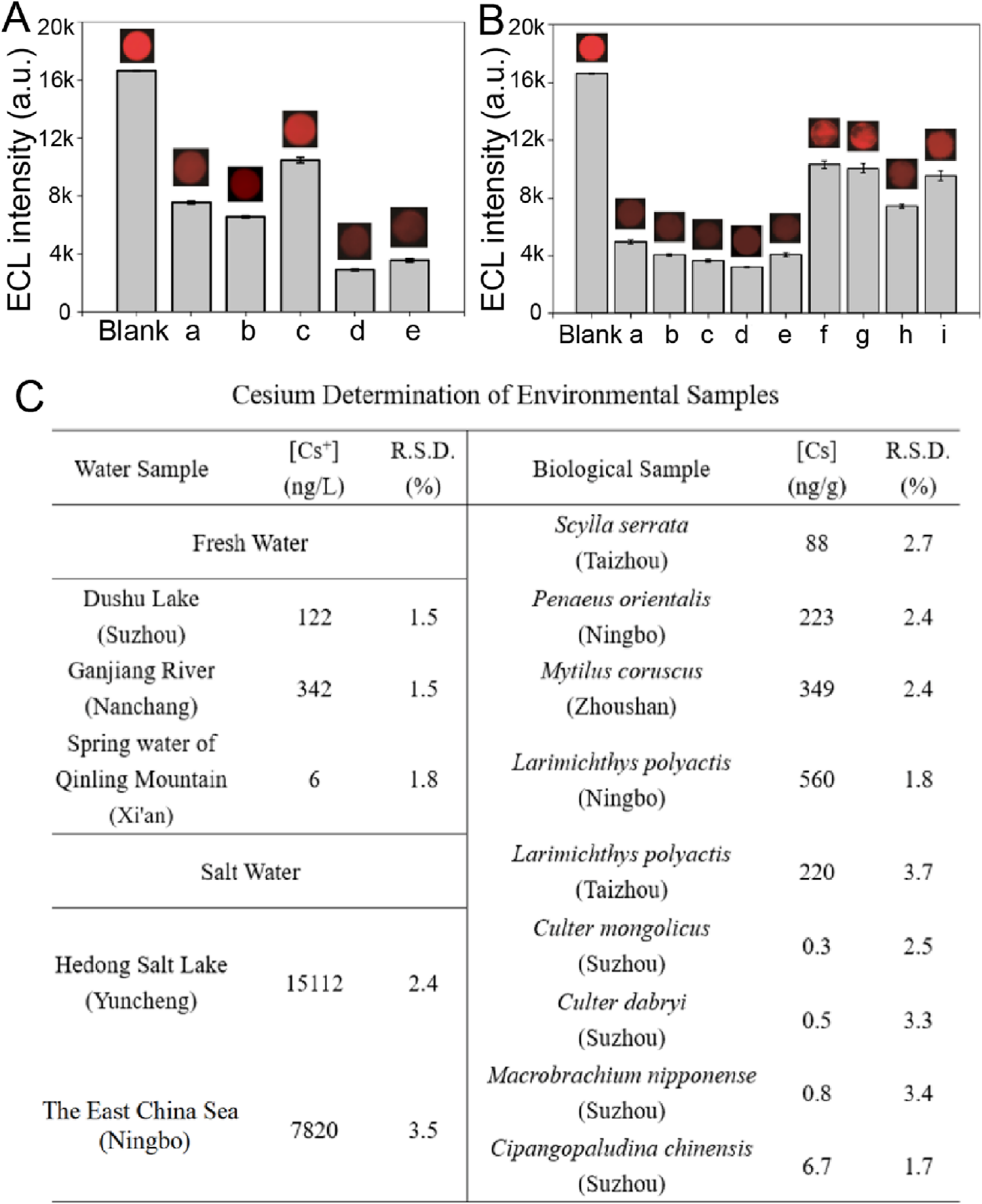
Cesium determination in practical samples. A) ECL signals and images of blank CIPF and CIPF treated with fresh water from (a) Dushu Lake, (b) the Ganjiang River, (c) spring water from Qinling Mountain as well as saltwater from (d) Hedong Salt Lake and (e) the East China Sea. B) Biological samples from the sea, (a) *Scylla serrata*, (b) *Penaeus orientalis*, (c) *Mytilus coruscus, and Larimichthys polyactis* from (d) Ningbo and (e) Taizhou. Biological samples from fresh water (Dushu Lake), (f) *Culter mongolicus*, (g) *Culter dabryi*, (h) *Cipangopaludina chinensis*, and (i) *Macrobrachium nipponense*. C) Detection results for environmental samples. Scan rate: 100 mV s^−1^ (400 mV s^−1^ for ECL imaging), PMT = 300 V, n = 3.

CIPF has been applied in the detection of various kinds of natural samples, including fresh water, saltwater, and biological samples (fish, arthropod, and mollusk) (**Figure** **6**). All experiments were conducted under the guidance of the National Research Council's Guide for the Care and Use of Laboratory Animals and approved by the Animal Ethics Committee of Soochow University (Permit Number: SUDA20240911A32). The locations of these samples and the photographs of the biological samples are shown in Figures S10 and S11 (Supporting Information), respectively. CIPF can be employed to visualize cesium in various kinds of environmental samples (such as salt water, fresh water as well as different kinds of animal samples, Figure 6A,B) and provides accurate determination results (Figure 6C). As a commonly used technology in heavy metal determinations, ICP‐MS is usually regarded as that it cannot be applied in the detection of ultra‐trace ions (lower than ng/L orders of magnitude) as well as samples with high‐salinity. Therefore, the environmental samples in Figure 6C cannot be measured by ICP‐MS directly. To confirm the correction of the measuring results by the ECL method, spiking determination experiment results are carried out in Figure S12 (Supporting Information) in which the actual results are similar to theoretical values. This result confirms the accuracy of ECL device in the determination of Cs^+^ in different samples. The cesium concentrations of saltwater samples (salt lake and seawater) are significantly higher than those of freshwater samples (Figure 6C). The cesium concentration of fresh water from lakes and rivers is also notably higher than that of spring water. This phenomenon may be attributed to the enrichment process from spring water to lakes and rivers and subsequently to saltwater lakes and seas. The cesium contents in marine animal samples (*Scylla serrata*, *Penaeus orientalis*, *Mytilus coruscus*, and *Larimichthys polyactis* collected from the East China Sea) are higher than those in the animal samples from freshwater (*Culter mongolicus, Culter dabryi, Cipangopaludina chinensis, and Macrobrachium nipponense* collected from Dushu Lake, Suzhou), which is similar to the results of water samples from the East China Sea and Dushu Lake. This may be due to the different Cs^+^ concentrations in their living environments.

**Figure 7:**
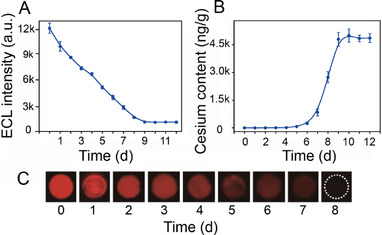
Cesium determination in *Misgurnus anguillicaudatus* samples and the newly designed ECL instrument. A) ECL signals of CIPF treated with samples of *Misgurnus anguillicaudatus* fed 100 mg L^−1^ Cs^+^ solution for different durations. Scan rate: 100 mV s^−1^, PMT = 300 V. B) Cesium content of *Misgurnus anguillicaudatus* samples. C) ECL images of CIPF treated with *Misgurnus anguillicaudatus* samples. Scan rate: 400 mV s^−1^, n = 3.

*Misgurnus anguillicaudatus* (a kind of miniature fish widely distributed in East Asia) was chosen as a model to perform a cesium stimulation experiment on wild fish to further demonstrate the practical application of this sensor in environmental determinations. After being fed with the cesium ion concentration of 100 mg L^−1^ for 10 d, a pronounced increase in the cesium content within the soft tissue was observed, and the final content was ≈4.5 µg g^−1^ (**Figure** **7**A,B). This result may be due to the cesium accumulation in the soft tissues of the animals. The ECL imaging results (Figure 7C) are similar to the results presented in Figure 7A, further confirming the reliability of this sensor in the visualized determination of cesium in practical environmental samples. The successful monitoring of cesium accumulation in animal samples demonstrates the application of this ECL system in ecology studies.

To achieve rapid and real‐time detection in practical environmental analysis, a portable and user‐friendly cesium detection system, including both a detection device and the newly designed instrument, has been developed. CIPF is further modified on screen‐printed electrodes to yield a new disposable chip‐type detection device due to its low cost (unit cost: 2–3 RMB), and an upsensing ECL detector is developed to match the device. The structures of the device and instrument as well as photographs of the practical instrument and detection device, are shown in Figures S13 and S14 (Supporting Information), respectively. The three‐electrode system is printed on the surface of the screen‐printed electrode. A matched chip‐type card slot is designed in the darkroom to insert the detection device, while the photomultiplier tube is installed on the upper side of the darkroom to make it suitable for the up‐emitting property of this device. Compared with the known technologies, this ECL detection system also exhibits obvious advantages in portability and simplification of pre‐treatment (Table S2, Supporting Information).

## 3 Conclusion

In summary, this work provides an efficient ion‐imprinted ECL detection system with a pore‐dependent ECL mechanism for the determination of trace cesium that is then successfully applied in the visualization of trace cesium in various types of environmental samples. In detail, porous poly(*o*‐phenylenediamine) was prepared on the surface of the electrodes to obtain an ECL sensor via an electrodeposition method with cesium‐matched pores. TPrA can enter these pores and be oxidized on the electrodes, while Ru(bpy)_3_^2+^ cannot. Cs^+^ binds to the ─N═ groups to occupy the Cs^+^‐matched pores and selectively block the oxidation process of TPrA in these pores. This process leads to an obvious ECL quenching of Ru(bpy)_3_^2+^ in PBS with an ultralow LOD value of 50 pg L^−1^. Good selectivity for cesium ions is also achieved because the Cs^+^ ions are well‐matched with the pores of CIPF. A chip‐type detection system is developed based on a screen‐printed electrode and an upsensing ECL instrument to realize the rapid determination of practical samples.

Compared with known cesium detection technologies (ICP‐MS, fluorescence, colorimetric, and electrochemical methods), this detection system achieves ultrahigh sensitivity, high salinity tolerance, and visualized determination of trace cesium, revealing the full potential of ECL technology for environmental monitoring. Therefore, this detection system has been successfully employed to the trace cesium determination in environmental samples (fresh water, saltwater, and different kinds of animals) and the cesium accumulation monitoring in animal samples, confirming its significance in environmental analysis and ecology studies. This ECL system is also expected to be extended to the multiplexed and accurate detection of various kinds of radioactive contaminants (such as radioactive iodide and strontium, uranyl ion, and so on) due to its potential adaptability to other target ions by tailoring imprinting monomers and templates.

## 4 Experimental Section

### Preparation of the Detection Device

The electropolymerization of *o*‐phenylenediamine (*o*‐PD) was carried out by CV from 0.2 to 0.8 V (50 mV s^−1^) in a conventional three‐electrode cell consisting of the surface of working electrodes (glassy carbon/screen printing electrodes). The polymerization solution used was PBS (0.1 mol L^−1^, pH 6.0) containing 5.0 mM *o*‐PD and 0.3 mM CsCl, and the Cs^+^‐binding poly(*o*‐phenylenediamine) film was obtained after 20 CV cycles. The obtained Cs^+^‐binding poly(*o*‐phenylenediamine) film was treated with 1 mol L^−1^ HCl for 3 h to remove the Cs^+^ templates and obtain CIPF. For the control experiments, a nonimprinted device was also fabricated via the same procedures, albeit without the addition of Cs^+^ during electropolymerization.

### ECL Measurement

Cs^+^ solutions with different concentrations were added to the Cs^+^ imprinting sensor and washed with deionized water after 30 min. 1 mM Ru(bpy)_3_Cl_2_ (ECL emitter) was diluted in 0.1 M pH 7.4 PBS to obtain an electrolyte. The selectivity measurements used Na^+^, K^+^, Ca^2+^, Mg^2+^, Sr^2+^, Mn^2+^, Cu^2+^, Zn^2+^, Pb^2+^, Ni^2+^, Fe^3+^, Al^3+^, Co^3+^, NH_4_^+^, Cl**^−^**, and SO_4_^2−^ as interference ions at a concentration of 250 mg L^−1^ for each ion. Acetic acid, oxalic acid, citric acid, lauryl sodium sulfate, polyethylene glycol, EDTA, glycine, and ciprofloxacin hydrochloride as interference organic acids, surfactants, and dissolved organic matters at a concentration of 250 mg L^−1^ for each matter. The detailed measurement procedure was the same as that used for Cs^+^ detection.

### Calculation Method of LOD Value

The LOD value is calculated through the equation:

(11) LOD=3δblank/s

In this equation, “δ_blank_” is the standard deviation of the ECL intensity value of the blank ECL device in 0.1 M PBS solution (pH 7.4) with 1 mM Ru(bpy)_3_Cl_2_ and 25 mM TPrA in Figure 5F, and “s” is the slope value in Figure 5B.

### DFT Calculations

All density functional theory (DFT) simulations were executed with the Gaussian 09 software package. A fragment model −C_6_H_4_ = N−C_6_H_4_−NH− (denoted as PoPD) containing the most important structural features was constructed to simulate the locally negatively charged structure of the imprinting structure. The geometries of PoPD and M^+^ (Na^+^, K^+^, and Cs^+^) and their complexes were optimized at the B3LYP Def2‐SVP level, and Grimme's empirical dispersion (GD3) correction was used to improve the description of van der Waals interactions. For single‐point energy calculations, higher‐precision calculations were performed on these optimized structures at the M062X/Def2‐TZVP SP level of theory. The electrostatic potential was calculated and displayed at an electron density isosurface. The binding energy (E_b_) is calculated as follows:

(12) $$E_{b}=E_{\mathrm{complex}\;}-\;E_{M}+-\;E_{\mathrm{Po}}\mathrm{PD}$$

where *E*_complex_, *E*_M+,_ and *E*_PoPD_ are the total energies of the total complex, M^+^ fragment, and poly(*o*‐phenylenediamine), respectively.

The materials and apparatus are listed in the Supporting Information. All animal experiments were conducted under the guidance of the National Research Council's Guide for the Care and Use of Laboratory Animals and were approved by the Animal Ethics Committee of Soochow University (Permit Number: SUDA20240911A32).

### Statistical Analysis

The detection data were expressed as mean±S.D. Cesium determination results were calculated by the equation listed in Figure 5B and carried out with the R.S.D. values for each one. The sample size (n) values for each cesium determination data are 3.

## Conflict of Interest

The authors declare no conflict of interest.

## Supporting information



Supporting Information

## Data Availability

The data that support the findings of this study are available from the corresponding author upon reasonable request.

## References

[1] K. O. Buesseler , Science 2020, 369, 621. 32764053 10.1126/science.abc1507

[2] J. McCurry , Lancet 2023, 402, 277. 37482063 10.1016/S0140-6736(23)01511-8

[3] F. Staeger , D. Zok , A. K. Schiller , B. Feng , G. Steinhauser , Environ. Sci. Technol. 2023, 57, 13601. 37646445 10.1021/acs.est.3c03565 PMC10501199

[4] R. W. Leggett , L. R. Williams , D. R. Melo , J. L. Lipsztein , Sci. Total Environ. 2003, 317, 235. 14630424 10.1016/S0048-9697(03)00333-4

[5] Y. Fakhri , M. Sarafraz , Z. Pilevar , H. Daraei , A. Rahimizadeh , S. Kazemi , K. M. Khedher , V. N. Thai , L. H. Ba , A. M. Khaneghah , Chemosphere 2022, 289, 133149. 34871618 10.1016/j.chemosphere.2021.133149

[6] Y. Fakhri , T. Mahmudiono , V. Ranaei , M. Sarafraz , A. Nematollahi , A. M. Khaneghah , Biol. Trace Elem. Res. 2023, 201, 2011. 35588038 10.1007/s12011-022-03289-1

[7] Y. Y. Zhao , H. Y. Sun , C. Wei , T. Y. Pan , L. Yang , M. L. Feng , X. Y. Huang , Adv. Funct. Mater. 2025, 35, 2425069.

[8] L. Cheng , S. Y. Li , Y. J. Zhang , X. Meng , Y. Wang , S. Wang , K. Y. Wang , Adv. Funct. Mater. 2025, 35, 2424406.

[9] L. Zhu , L. Zhu , T. Shi , K. Zhao , W. He , L. Han , Z. Jin , J. Kang , S. Sun , N. Cao , Z. Yu , Adv. Sci. 2025, 12, 2505997. 10.1002/advs.202505997 PMC12302566 40349164

[10] V. N. Epov , D. Lariviere , K. M. Reiber , R. D. Evans , R. J. Cornett , J. Anal. At. Spectrom. 2004, 19, 1225.

[11] D. Karamanis , P. A. Assimakopoulos , Water Res. 2007, 41, 1897. 17374545 10.1016/j.watres.2007.01.053

[12] J. Fu , L. Zhang , S. L. Wang , W. L. Yuan , G. H. Zhang , Q. H. Zhu , H. Chen , L. He , G. H. Tao , J. Hazard. Mater. 2022, 425, 127981. 34883380 10.1016/j.jhazmat.2021.127981

[13] Y. C. Yang , J. P. Hsu , J. Phys. Chem. C 2021, 125, 24211.

[14] R. Umapathi , C. V. Raju , M. Safarkhani , J. Haribabu , H. U. Lee , G. M. Rani , Y. S. Huh , Coord. Chem. Rev. 2025, 525, 216305.

[15] J. Qiu , L. Fu , H. Wang , R. Zou , Y. Zhang , X. Li , A. Wu , New J. Chem. 2020, 44, 2241.

[16] G. Jia , J. Jia , Appl. Radiat. Isot. 2024, 209, 111338. 38714137 10.1016/j.apradiso.2024.111338

[17] J. M. Wong , R. Zhang , P. Xie , L. Yang , M. Zhang , R. Zhou , R. Wang , Y. Shen , B. Yang , H. B. Wang , Z. Ding , Angew. Chem., Int. Ed. 2020, 59, 17461. 10.1002/anie.202007588 32588510

[18] S. Knezevic , D. Han , B. Liu , D. Jiang , N. Sojic , Angew. Chem., Int. Ed. 2024, 63, 202407588. 10.1002/anie.202407588 38742673

[19] J. Descamps , C. Colin , G. Tessier , S. Arbault , N. Sojic , Angew. Chem., Int. Ed. 2023, 62, 202218574. 10.1002/anie.202218574 36811514

[20] C. Ma , Y. Cao , X. Gou , J. J. Zhu , Anal. Chem. 2020, 92, 431. 31679341 10.1021/acs.analchem.9b04947

[21] W. Miao , Chem. Rev. 2008, 108, 2506. 18505298 10.1021/cr068083a

[22] J. Dong , Y. Lu , Y. Xu , F. Chen , J. Yang , Y. Chen , J. Feng , Nature 2021, 596, 244. 34381236 10.1038/s41586-021-03715-9

[23] S. A. Kitte , F. A. Bushira , C. Xu , Y. Wang , H. Li , Y. Jin , Anal. Chem. 2022, 94, 1406. 34927425 10.1021/acs.analchem.1c04726

[24] W. R. Cui , C. R. Zhang , W. Jiang , F. F. Li , R. P. Liang , J. Liu , J. D. Qiu , Nat. Commun. 2020, 11, 436. 31974343 10.1038/s41467-020-14289-x PMC6978342

[25] Z. Wang , Z. Zhao , Y. Pei , Y. Xia , F. Chen , M. Xu , H. Gao , D. Hua , Sens. Actuators, B 2023, 395, 134506.

[26] Z. Wang , Y. Li , J. B. Pan , M. Xu , J. J. Xu , D. Hua , J. Hazard. Mater. 2023, 453, 131449. 37086673 10.1016/j.jhazmat.2023.131449

[27] Z. Wang , J. B. Pan , Q. Li , Y. Zhou , S. Yang , J. J. Xu , D. Hua , Adv. Funct. Mater. 2020, 30, 2000220.

[28] Y. B. Amram , R. Tel‐Vered , M. Riskin , Z. G. Wang , I. Willner , Chem. Sci. 2012, 3, 162.

[29] J. D. Chaplin , P. E. Warwick , A. B. Cundy , F. Bochud , P. Froidevaux , Anal. Chem. 2021, 93, 11937. 34432435 10.1021/acs.analchem.1c01342

[30] S. Shinde , M. Mansour , A. Incel , L. Mavliutova , C. Wierzbicka , B. Sellergren , Chem. Sci. 2020, 11, 4246. 10.1021/acsomega.1c05079 PMC8757333 35036726

[31] T. C. Pereira , N. R. Stradiotto , Microchim. Acta 2019, 186, 764. 10.1007/s00604-019-3898-3 31713083

[32] Y. Yuan , Y. Yang , X. Ma , Q. Meng , L. Wang , S. Zhao , G. Zhu , Adv. Mater. 2018, 30, 1706507. 10.1002/adma.201706507 29423920

[33] A. V. Klimashevskaya , K. V. Arsenyeva , A. V. Cherkasov , I. A. Yakushev , P. V. Dorovatovskii , A. V. Piskunov , J. Struct. Chem. 2023, 64, 2271.

[34] J. Mahler , I. Persson , Inorg. Chem. 2012, 51, 425. 22168370 10.1021/ic2018693 PMC3250073

[35] M. M. Richter , Chem. Rev. 2004, 104, 3003. 15186186 10.1021/cr020373d

[36] M. E. Moret , I. Tavernelli , U. Rothlisberger , J. Phys. Chem. B 2009, 113, 7737. 19435301 10.1021/jp900147r

[37] W. Miao , J. P. Choi , A. J. Bard , J. Am. Chem. Soc. 2002, 124, 14478. 12452725 10.1021/ja027532v

[38] Z. Xing , X. Lu , Z. Zhang , Y. Zhao , Y. Cao , Y. Zhou , J. J. Zhu , Adv. Funct. Mater. 2025, 35, 2425768.

[39] P. Zhu , Y. Zhao , Mater. Chem. Phys. 2019, 233, 60.

[40] Y. Huang , J. Wan , T. Pan , K. Ge , Y. Guo , J. Duan , J. Bai , W. Jin , S. Kitagawa , J. Am. Chem. Soc. 2023, 145, 24425. 37880205 10.1021/jacs.3c10277

[41] R. R. Sharma , S. Chellam , Environ. Sci. Technol. 2005, 39, 5022. 16053106 10.1021/es0501363

