# Supplementary material for: Ultrasensitive Ion‐Imprinted Detection System with Pore‐Depended Electrochemiluminescence Mechanism for Accurate and Rapid Monitoring of Cesium in the Environment
Source: Adv Sci (Weinh). 2025 Oct 30;13(4):e16113. doi: 10.1002/advs.202516113 (PMC12822453; doi:10.1002/advs.202516113)
Supplement: Supplementary file 1 — Supporting Information [file ADVS-13-e16113-s001.docx]

Supporting Information

Ultrasensitive Ion-imprinted Detection System with Pore-depended Electrochemiluminescence Mechanism for Accurate and Rapid Monitoring of Cesium in the Environment

Ziyu Wang, Lei Fang, Jianing Zhao, Chengqi Li, Hebing Xie, Jian-Bin Pan, Daoben Hua*

E-mail: dbhua_lab@suda.edu.cn

**Content**

1. Materials and apparatus …………………………………………………………………... S2

2. ECL spectrum measurement ……………………………………………………………… S2

3. Calculation method of LOD value ………………………………………………………… S3

4. Calculation of imprinting pores …………………………………………………………… S3

5. Calculation of electrochemical active surface area (ECSA) values ……………………… S3

6. Calculation of hydrated diameter of ions ………………………………………………… S3

7. Environmental Sample Pretreatment ………...…………………………………………… S3

8. Calculation method of ECL efficiency ……...………………………………….………… S4

9. The selectivity coefficient and relative selectivity coefficient.………………….………… S4

10. The LODs of Cs^+^ detection methods in standards and previous literature …………….… S5

11. Comparison of the portability and pre-treatment methods detection instruments……….. S6

12. CV data and ECL intensity values of electropolymerization …….…………………...… S7

13. DFT calculation …….……………………………………………………………….…... S7

14. ECL spectrum and signal of Ru(bpy)_3_Cl_2_ of TPrA …….……………………………...… S8

15. Supplement to the response mechanism …….……………………………………..….… S8

16. Long-term stability of the probe …….……….………………………………………….. S9

17. Electrochemical impedance spectra …….……………………………………………..… S9

18. ​​Ion adsorption results​ …….……………………………………………………………... S9

19. TEM images and the average size of CIPF at different temperature …….……………… S10

20. Locations of practical samples …….…………………………………………………… S10

21. Photographs of animal samples …….……………………………………………..…..…S11

22. Results of spiking experiments of practical samples ……………………………………S11

23. Structure of newly designed SPE based up-sensing ECL detector …………..……….…S12

24. Photographs of newly designed instrument ………………………………………..….…S12

25. Reference ……………………………………………………………………………..…S13

**Materials and apparatus.** All solvents and reagents were commercially available and of analytical reagent grade. 0.1 M Phosphate buffered saline (PBS) was purchased from Jiangsu KeyGEN Bio TECH Co., Ltd (Nanjing, China). Glassy carbon electrode (GCE), silver chloride electrode (Ag/AgCl) and Platinum wire electrode were purchased from Tianjin Incole Union Tecology Co., Ltd (Tianjin, China). ITO conductive film glass electrode was purchased from Xiangcheng South China Technology Co., Ltd. (Shenzhen, China). 1,2-Phenylenediamine (99%, RG) were purchased from Shanghai Titan Scientific Co., Ltd (Shanghai, China). CsCl were purchased from Sigma-Aldrich Co., Ltd (Shanghai, China). Tripropylamine were purchased from Sinopharm Chemical Reagent Co., Ltd (Shanghai, China). Double-distilled water was used throughout the experiments. The animal samples from fresh water (*Culter mongolicus*, *Culter dabryi*, *Cipangopaludina* *chinensis* and *Macrobrachium nipponense*) were collected from Dush Lake (Suzhou, China) by using lure fishing method and fishing cages. The marine animal samples (*Scylla serrata*, *Penaeus orientalis*, *Mytilus coruscus* and *Larimichthys polyactis*) were supplied by Zhejiang Provincial Center for Disease Control and Prevention, which are corrected in August, 2023. All experiments were conducted under the guidance of National Research Council’s Guide for the Care and Use of Laboratory Animals and approved by the Animal Ethics Committee of Soochow University (Permit Number: SUDA20240911A32). Electrochemical and ECL spectra were conducted on an ECL analyzer equipped with a CHI-660E electrochemical station supplied by the State Key Laboratory of Analytical Chemistry for Life Science, Nanjing University. ECL emission measurements were conducted on a self-developed ECL analyzer supplied by the State Key Laboratory of Analytical Chemistry for Life Science, Nanjing University. The spectral width of the photomultiplier tube (PMT) was 350-650 nm. X-ray photoelectron spectroscopy was measured by Feynman Biotechnology Tech Co., Ltd (Suzhou, China). TEM images were taken by **High-Resolution Transmission Electron Microscope with FEG, Talos F200S G2.** The concentration of ions in Figure S7 were determined by inductively coupled plasma mass spectrometer (ICP-MS, Agilent 7850), and the measurements are entrusted to the Scientific Compass Tech Co., Ltd (Zhejiang, China).

**ECL spectrum measurement.** The ECL spectra were measured using CHI 660E electrochemical workstation in conjunction with a steady-state transient fluorescence spectrometer (Edinburgh Instruments, FLS980) in the closed state of lamp. An ITO electrode with cesium-binding poly(*o*-phenylenediamine), a CIPF-modified ITO electrode, and a CIPF-modified ITO electrode treated with 1 ng/L Cs⁺ solution were used as working electrodes, Ag/AgCl and Pt wire served as the reference electrode and counter electrode, respectively. Experiments were performed in PBS solution (0.1 mol/L, pH 7.4) containing 1 mM Ru(bpy)₃Cl₂ by applying ranging from 1.2 to 2.0 V potentials for 20 seconds with a scan rate of 200 mV/s, their ECL spectra were recorded with 2 nm slit width from 500 to700 nm. A quartz cuvette (3.5 mL) with 10 mm path length was used as an electrochemical cell.

**Calculation of imprinting pores.** TEM is applied to scan the sample surface and obtain surface image. Pore size distribution analysis was performed using TEM micrographs of CIPF processed in ImageJ (NIH, USA). The 10-nm scale bar was calibrated (Analyze > Set Scale), 100 randomly selected well-defined pores were measured and exported to CSV.

**The calculation of electrochemical surface area (ECSA) values.** Glassy carbon electrode electropolymerized by *o*-phenylenediamine and treated by various treatment methods which in 5 mM K_3_Fe(CN)_6_ solution (containing 0.1 M PBS as supporting electrolyte) cyclic voltammetry curves at 0.1 V/s scan rates. Under semi-infinite diffusion-controlled conditions at a room temperature of 25°C, the relationship between the peak current value (*i*_p_) and the square root of the scan rate (*v*^1/2^) can be described by the Randles-Sevcik equation:

$$i_{p}=\left( 2.69\times{10}^{5} \right)n^{3/2}AD^{1/2}Cv^{1/2}$$

In this formula, *i*_p_ is the peak current value of oxidation wave in the CV data (A); *n* is the number of electrons transferred in the redox event; *A* is the electrode area (cm^2^); *C* is the concentration of K_3_Fe(CN)_6_ in the electrolyte (mol/cm^3^); *v* is the scan rate (V/s). *D* is the diffusion coefficient of ions (cm^2^/s) can be calculated from the formula.

**Calculation of hydrated diameter of ions**. The hydrated diameter values of metal ions (M^+^: Na^+^, K^+^, and Cs^+^) are calculated by using the data in previous literature (*Inorg. Chem.* **2012**, *51*, 425) in aqueous solution at room temperature using the formula: r/Å = d_M-O_/Å + r_O_/Å. In this formula, r/Å, d_M-O_/Å and d_O_/Å are the hydrated radius of alkali metal ions, M-O bond distances in the hydrated alkali metal ions (d_Na-O_, d_K-O_ and d_Cs-O_ is 2.43, 2.80, and 3.07 Å) and radius of oxygen (1.34 Å). The hydrated diameter value is 2r/Å.

**Environmental** **Sample Pretreatment:** The water samples are filtered by using 0.22 μm filter membrane. For the biological samples, after euthanasia, the middle muscle tissues were dissected using sterile tools, weighed precisely, and placed in 50 mL sample bottles. Thirty milliliters of freshly prepared aqua regia (1:3 volume ratio of concentrated nitric acid to hydrochloric acid) were added to each sample bottle to ensure complete immersion of the tissue. The bottles were sealed with closure membranes and left at room temperature (25 ± 5°C) for 48 hours, until the solution became transparent. After digestion, the solution was carefully transferred to clean 50 mL conical flasks, and the sample identification number was labeled on the flasks. The conical flasks were placed on a temperature-controlled ultrasonic heating platform. The temperature was initially set to approximately 280°C to evaporate most of the solution, then raised to 310°C to further evaporate until the solution reached a near-dry state (volume reduced to 0.5-1 mL). The resulting solution was colorless or pale yellow and free from noticeable acid fumes, ensuring the complete destruction of the organic matrix and removal of residual acids. The concentrated solution was allowed to cool to room temperature. The conical flasks were rinsed multiple times with ultrapure water, and the solution was filtered through a 0.22 μm water-based micropore filter to remove any remaining particulates. The filtered solution was transferred to a 10 mL volumetric flask and diluted to the 10.0 mL mark with ultrapure water, ensuring uniform mixing. Depending on the mass of the samples, the prepared solution was further diluted with ultrapure water to achieve a final concentration of 0.02 mg/L for subsequent analysis.

**Calculation method of ECL efficiency.** The relative ECL efficiency value can be calculated through the following formula:


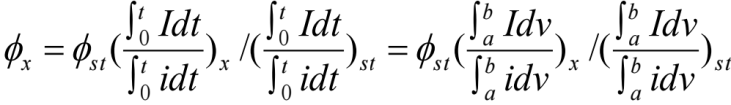


In this formula, *Ø*st is the ECL efficiency of the Ru(bpy)_3_Cl_2_ (1 mM)/TPrA (25 mM) system in a 0.1 M PBS solution with pH = 7.4, which is regarded as 100%. *I* is regarded as the ECL intensity value, *i* is regarded as the current value, x is regarded as the sample.

**The selectivity coefficient and relative selectivity coefficient.** The adsorption experiment to investigate the relative selectivity coefficient was performed in a multicomponent cation solution, where the concentration of each ion (Cs⁺ and the typical interference ion K⁺) was 10 μg/L. The CIPF/poly(*o*-phenylenediamine) film electropolymerized onto glassy carbon electrodes were immersed in 10 mL of the mixed ionic solution for 3 h. The concentrations of Cs⁺ and K⁺ in the solutions before and after adsorption were measured using ICP-MS.

The distribution coefficient (K_d_) of CIPF toward ions can be calculated according to formula^[1]^:

$$K_{\text{d}}=\frac{\left( C_{0}-C_{\text{e}} \right)V}{mC_{\text{e}}}$$

where *C*_0_ (μg/L) is the initial ions concentration, *C*_e_ (μg/L) is the equilibrium ions concentration, V (L) is the volume of the test solution, and m (g) is the mass of the adsorbent. Then can calculate the selectivity coefficient *β* (K_d_(Cs^+^)/K_d_(K^+^)) and relative selectivity coefficient K_d_’ (*β*_CIPF_/*β*_poly(o-phenylenediamine)_ _film_), *β*_CIPF_ and *β*_poly(o-phenylenediamine) film_ represent the selectivity coefficients of CIPF and poly(*o*-phenylenediamine) film, respectively.

**Table S1.** LOD values of chemical probes in previous studies.

| **Cs^+^ Probes** | **LODs** |
| --- | --- |
| Doubly crowned 1,3-calix[4]arenes PVC membrane electrodes ^[2]^ | 39.9 μg L^-1^ |
| Anilino-(1,3-dioxo-2-indanylidene) acetonitrile derivatives PVC membrane electrodes^[3]^ | 837.9 μg L^-1^ |
| Lipophilic calix[6]arene tetraester derivatives PVC membrane electrodes^[4]^ | 65.17 μg L^-1^ |
| Pyrone compound electrodes^[5]^ | 664.5 μg L^-1^ |
| Calix[4]arene-crown-6 derivatives fluorescent probe^[6]^ | 13.3 μg L^-1^ |
| 16-membered macrocyclic diamide PVC membrane electrodes^[7]^ | 625.1 μg L^-1^ |
| Employing calix[4]crown ether–ester PVC membrane electrodes^[8]^ | 665 μg L^-1^ |
| Potentiometric sensor PTEV-based zeolite membrane^[9]^ | 10640 μg L^-1^ |
| Cs‐12‐molybdophosphate PVC membrane electrodes^[10]^ | 399 μg L^-1^ |
| Ethylene glycol-functionalized microspheres ISE^[11]^ | 666 μg L^-1^ |
| Crown bridged thiacalix 4 arenes PVC membrane electrodes ^[12]^ | 13.3 μg L^-1^ |
| Cavitand PVC membrane electrodes ^[5]^ | 665 μg L^-1^ |
| DOS-plasticized PVC electrodes ^[13]^ | 1.05 μg L^-1^ |
| 15-crown-5 derivative fluorescent probe ^[14]^ | 399 μg L^-1^ |
| Zeolite-Modified Sol-Gel Electrode ^[15]^ | 970.9 μg L^-1^ |
| calix [4]arene-crown-6 compounds electrodes ^[16]^ | 11.27 μg L^-1^ |
| 15-Crown-5-Anthracene optode membrane fluorescent probe ^[17]^ | 5586 μg L^-1^ |
| Calix[4]arene-crown-6 PVC liquid membrane ISE ^[16]^ | 1.127 μg L^-1^ |
| Squaraine based quenching fluorescence probe ^[18]^ | 12.76 μg L^-1^ |
| Dibenzo-24-crown-8 complex IIP ^[19]^ | 0.7 μg L^-1^ |
| G-pentaplex-based luminescent Ir(III) complex ^[20]^ | 3325μg L^-1^ |
| CG chemo-indicator paper-based colorimetric sensor ^[21]^ | 100 μg L^-1^ |
| Calix[6]arene derivative optode membrane ^[22]^ | 1330 μg L^-1^ |
| Calix-COU-Benz-CN based fluorescent probe ^[23]^ | 186.2 μg L^-1^ |
| BTA-based chromogenic probe ^[24]^ | 44950 μg L^-1^ |
| Calix[4]arene biscrown-6 based fluorescent probe ^[25]^ | 93.1 μg L^-1^ |
| PDA vesicle-based colorimetric sensor ^[26]^ | 622.4 μg L^-1^ |
| AuNPs-PB based colorimetric sensor ^[27]^ | 2527 μg L^-1^ |
| Fe_3_O_4_@SiO_2_ IIP potentiometric microsensor ^[28]^ | 39.9 μg L^-1^ |
| Sumanene derivative fluorescent probe ^[29]^ | 19.95 μg L^-1^ |
| MMWCNTs@Cs(I)-IIP potentiometric sensor ^[30]^ | 5.32 μg L^-1^ |
| Ultralow-cost portable device perovskite fluorescence probe ^[31]^ | 180 μg L^-1^ |
| Ion exchange EG-OFET sensor ^[32]^ | 0.26 ng L^-1^ |
| π-extended and sumanene–ferrocene conjugates electrochemical sensor ^[33]^ | 6.65 μg L^-1^ |
| This work | 50 pg L^-1^ |

**Table S2.** Comparison of the portability and pre-treatment methods for detection instruments.

| **Technique** | **Portability profile** | **Sample pretreatment profile** |
| --- | --- | --- |
| This work | Portable instrument, disposable detection device | Direct detection |
| Radiochemical Analysis | Large and fixed labs, bulky instruments^[34]^ | Multi-step purification^[34]^ |
| Inductively coupled plasma-Mass Spectrometry | High-power/temperature systems, bulky instruments, shielding gas required^[35]^ | Multi-step sample preparation to combat matrix effects^[36]^ |
| Atomic absorption spectroscopy | High-power/temperature systems, bulky instruments, shielding gas required^[37]^ | Pre-treatments required such as concentration and enrichment^[38]^ |
| Ion chromatography | Bulky high-pressure flow path and separation system, limits miniaturization^[39]^ | Pre-treatment column needed^[40]^ |


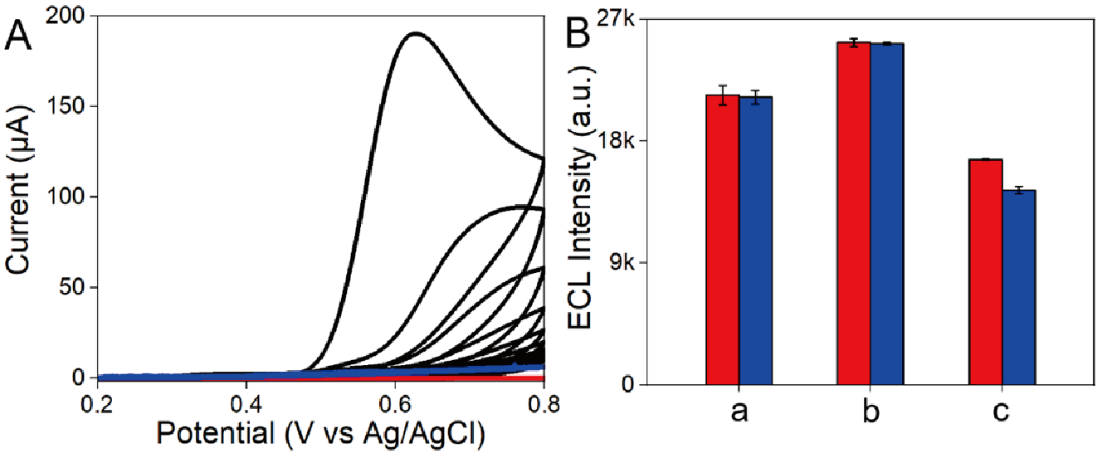


**Figure S1.** (A) CV data of electrodeposition process of poly(*o*-phenylenediamine) in deionized water (red), deionized water with 0.3 mM Cs^+^ (blue) and 0.1 M PBS solution (pH = 7.4) with 0.3 mM Cs^+^ (black). (B) (a) ECL intensity values of CIPF prepared in 10 mM Cs^+^, (b) 100 mM Cs^+^, and (c) 0.1 M PBS with 0.3 mM Cs^+^. Scan rate: 100 mV/s, PMT = 300 V.

(In the preparation process of Cs^+^ combined poly(*o*-phenylenediamine) film, only the one prepared in PBS solution with 0.3 mM Cs^+^ exhibits obvious current (Figure S1A). The CIPF prepared by 10 and 100 mM CsCl cannot give an obvious response to 100 pg/L Cs^+^ solution compared with CIPF prepared in 0.1 M PBS with 0.3 mM Cs^+^, indicating their poor sensitivity (Figure S1B). It can probably be attributed to the excessive Cs^+^ selective pores produced by the much higher Cs^+^ concentration in electrodeposition process. Considering to the poor electrical conductivity of 0.3 mM Cs^+^ solution (Figure S1A), the 0.1 M PBS solution is necessary in the preparation of CIPF.)


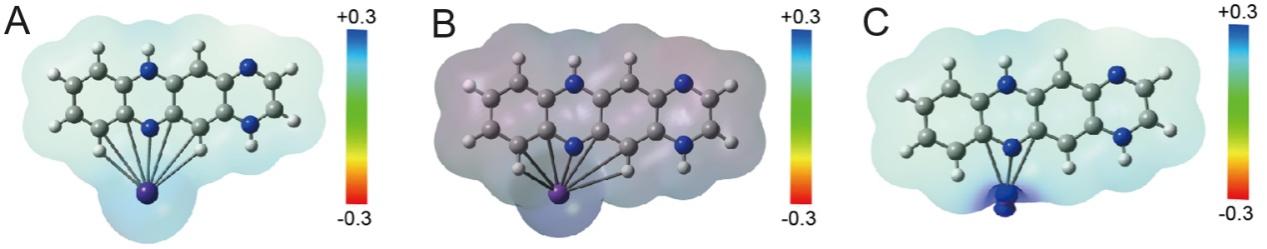


**Figure S2.** Electrostatic potential energy of -N= group with (A) Cs^+^, (B) K^+^ and (C) Na^+^.


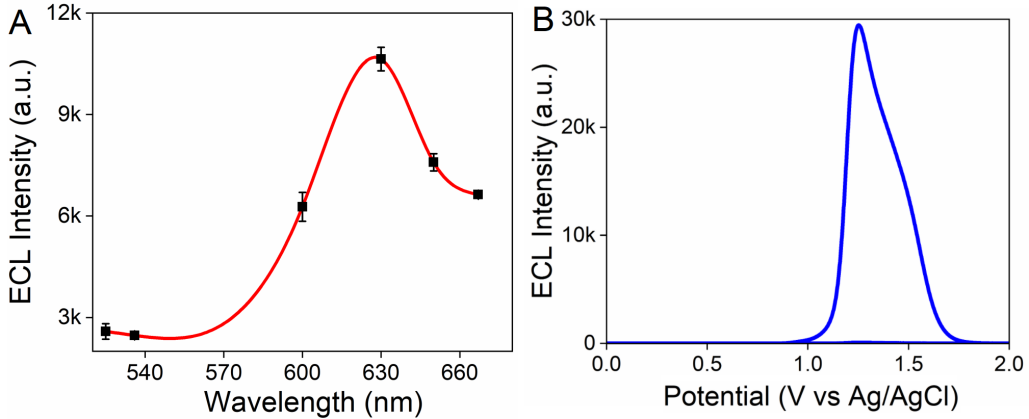


**Figure S3.** (A) ECL spectrum and (B) ECL signal of Ru(bpy)_3_Cl_2_ in 0.1 M PBS solution with 25 mM TPrA. Scan rate: 100 mV/s, PMT = 300 V.


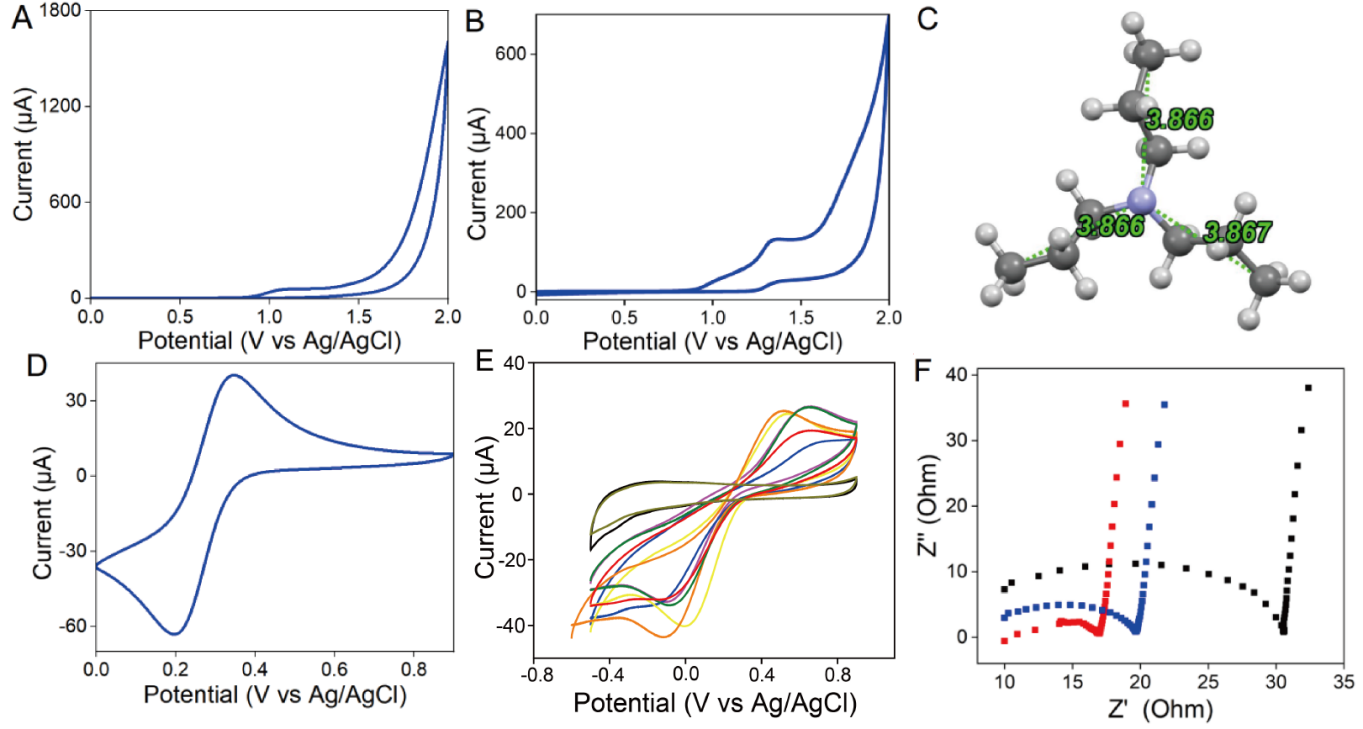


**Figure S4.** CV of (A) 25 mM TPrA and (B) 1 mM Ru(bpy)_3_Cl_2_ in 0.1 M PBS solution (pH = 7.4). (C) Molecular size of TPrA. CV of (D) 0.5 M Na_2_SO_4_ with 5 mM K_3_Fe(CN)_6_ with the working electrode as blank electrode; and (E) electrodes modified by cesium combined poly(*o*-phenylenediamine) (black), poly(*o*-phenylenediamine) (brown), CIPF (yellow), CIPF treated with 10 ng/L Cs^+^ (red), 10 μg/L Cs^+^ (blue), 40 mM Na^+^ (green), 40 mM K^+^ (purple) under room temperature, and CIPF treated with 10 μg/L Cs^+^ under 100 ^o^C (orange). (F) Electrochemical impedance spectra of cesium-binding poly(*o*-phenylenediamine) film (black), CIPF (red), and CIPF treated with 1 ng/L Cs^+^ solution (blue) in 0.1 M PBS solution (pH = 7.4) with 1 mM Ru(bpy)_3_Cl_2_ and 25 mM TPrA. Scan rate: 100 mV/s, PMT = 300 V.


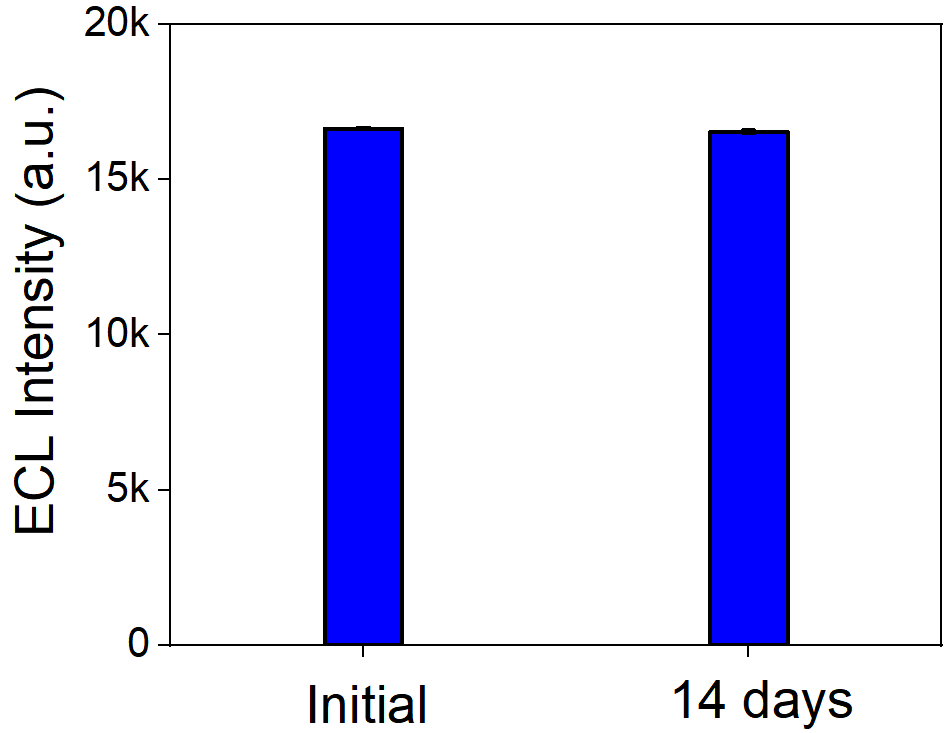


**Figure S5.** ECL intensity of ECL device before and after storage for 14 days. Scan rate: 100 mV/s, PMT: 300 V.


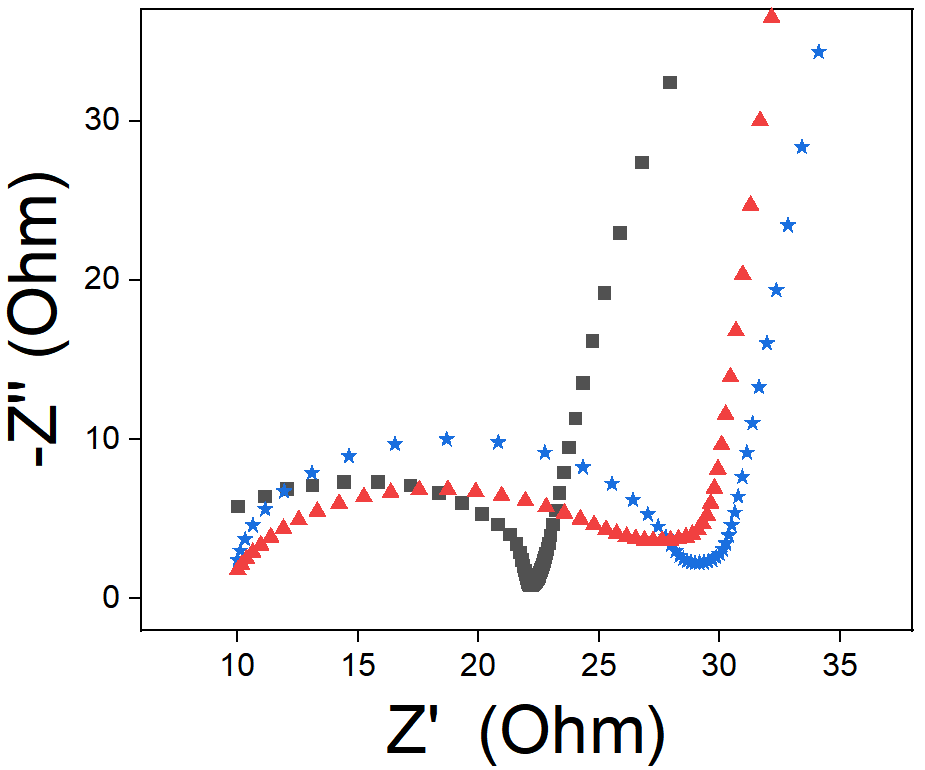


**Figure S6.** Electrochemical impedance spectra of blank poly(*o*-phenylenediamine) (blue) and poly(*o*-phenylenediamine) eluted by 1 M HCl (red) as well as CIPF (black).


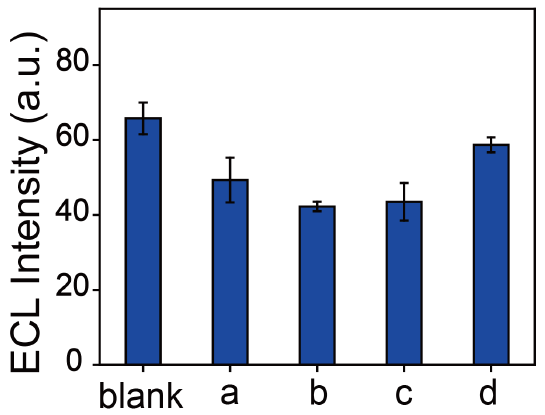


**Figure S7**. Enlarged ECL signals of the poly(*o*-phenylenediamine) film in Figure 5F.


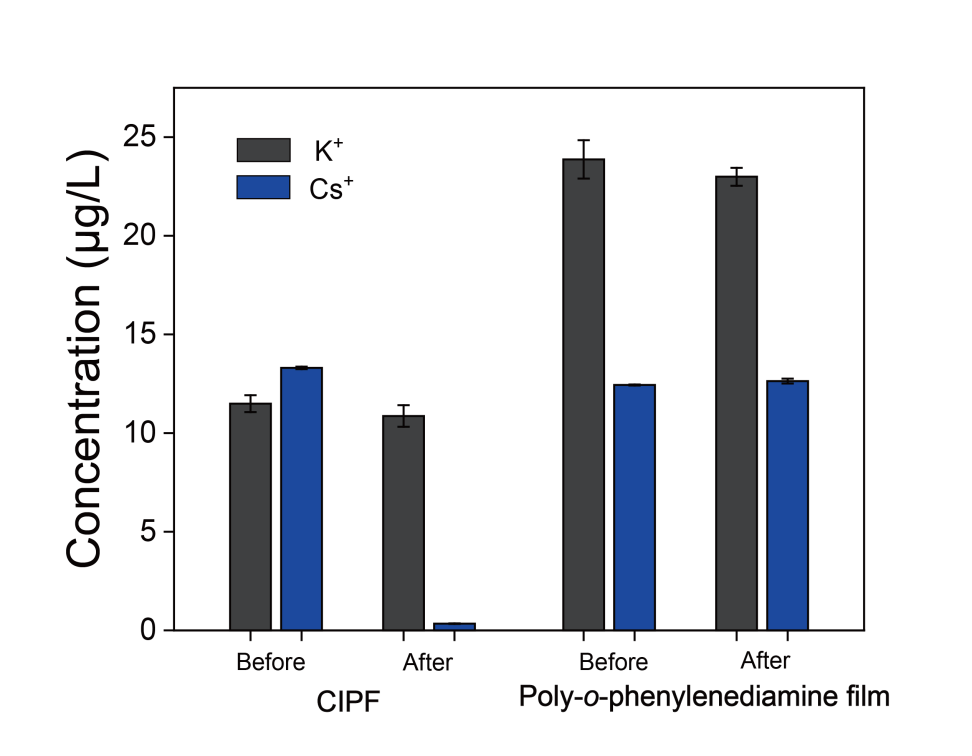


**Figure S8**. Concentrations (measured by ICP-MS) of K⁺ (black) and Cs⁺ (blue) before and after adsorption by the CIPF and poly(*o*-phenylenediamine) films electropolymerized onto glassy carbon electrodes.


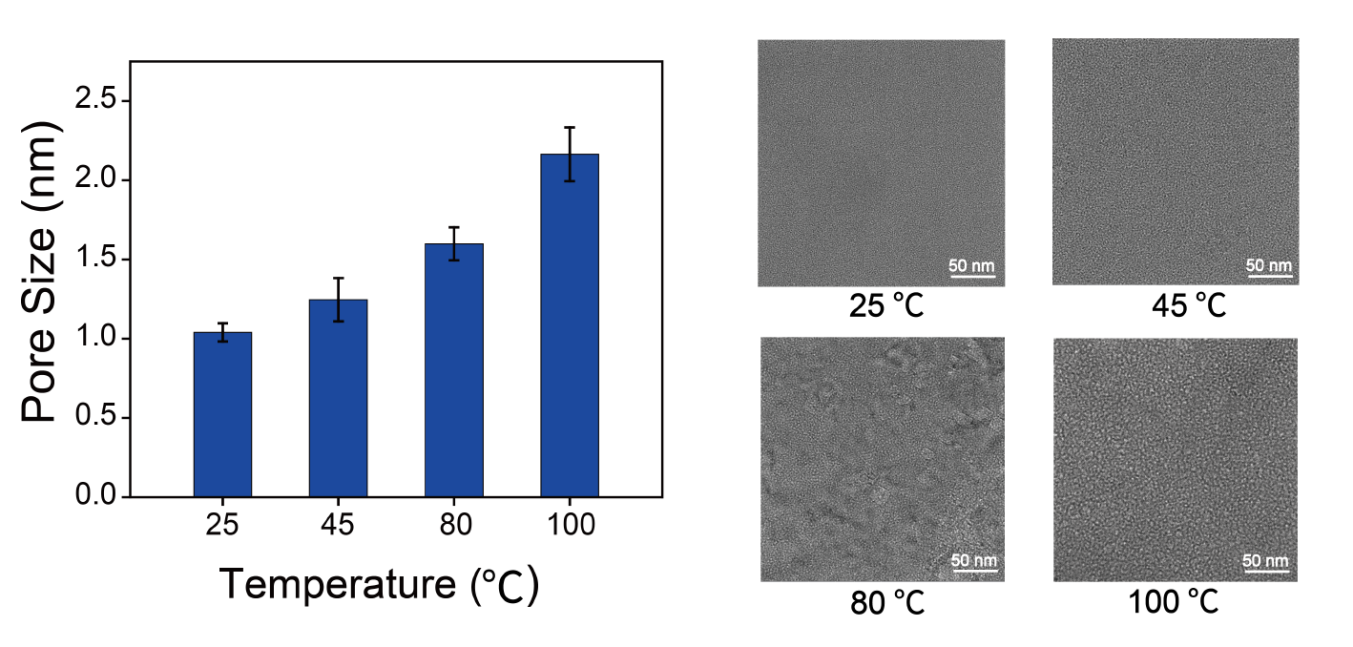


**Figure S9.** TEM images and the average size of CIPF at different temperature.


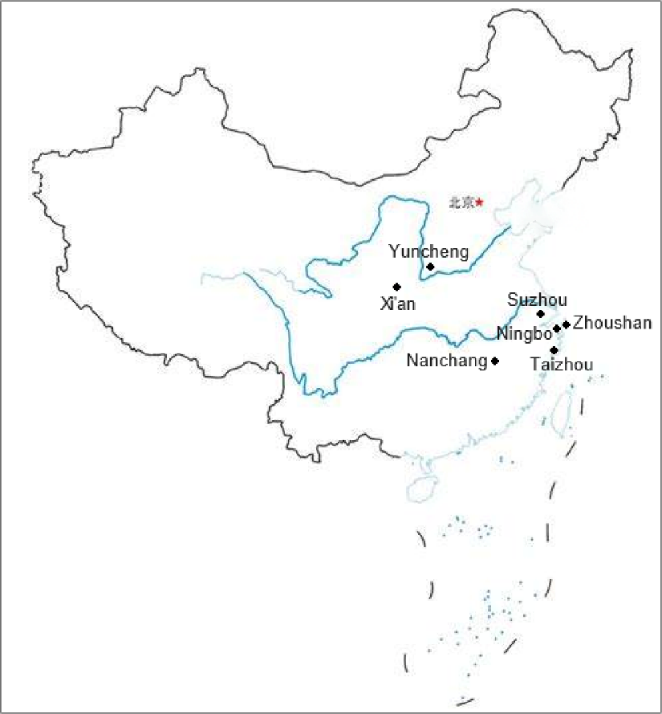


**Figure S10.** Locations of environmental samples.


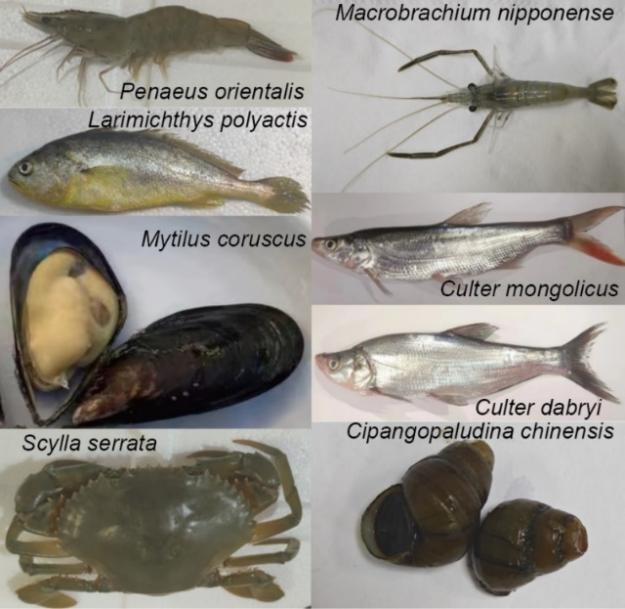


**Figure S11.** Photographs of animal samples.


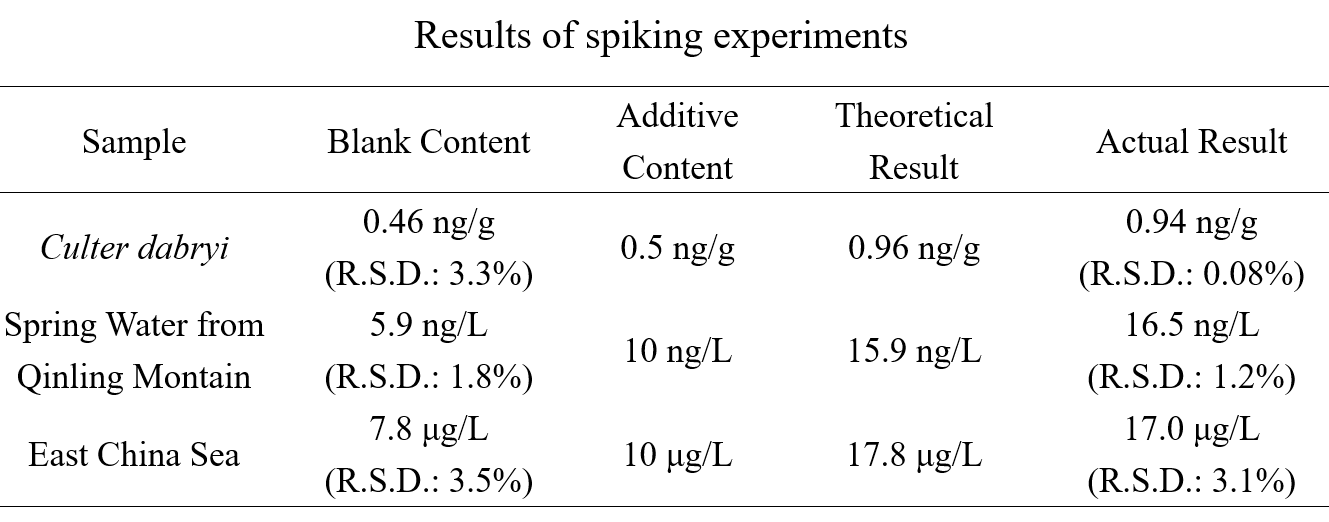


**Figure S12.** Results of spiking experiments of practical samples.


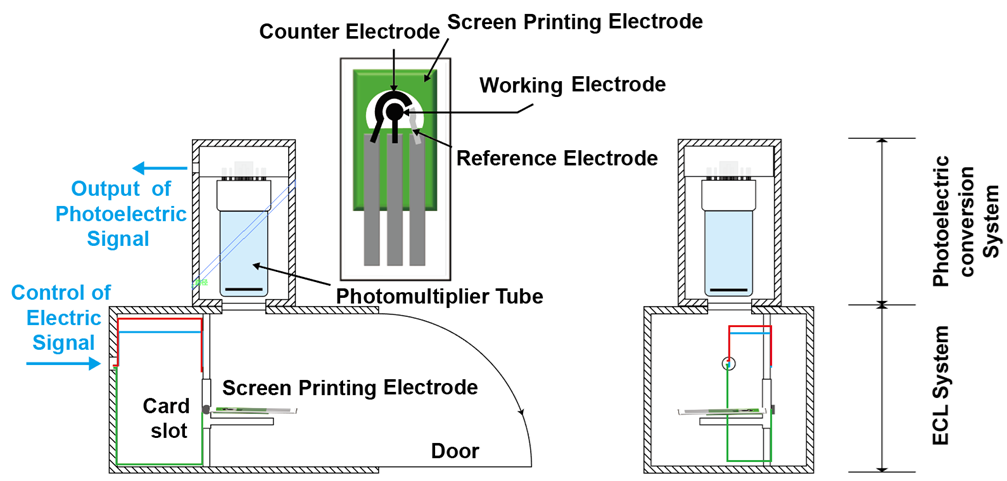


**Figure S13.** Structure of newly designed SPE based up-sensing ECL detector.


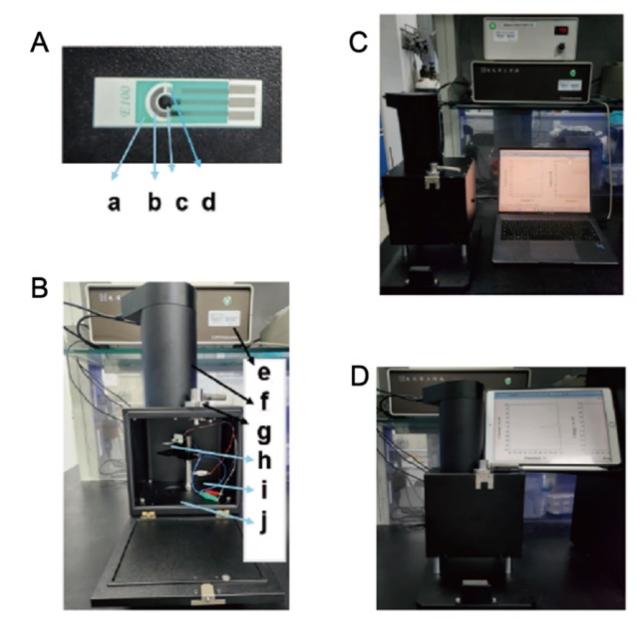


**Figure S14.** The structure and practical application photographs of detection device and chip-type instrument. (A) Detection devices with (a) insulating layer, (b) counter electrode, (c) working electrode, and (d) reference electrode. (B) Newly designed ECL detector with (e) controller, (f) PMT, (g) lock, (h) measuring pool, (i) multi-purpose electrode clamp, and (j) darkroom. (C) The practical application of this instrument in ECL detection using ultrabook. (D) The practical application of this instrument in ECL detection using bluetooth-connected tablet computer.

**References**

[1] W. Wang, M. Xu, H. Wu, Y. Song, P. Liu, H. Yu, L. Zhang, S. Chen, D. Hua, *Adv. Sci.* **2024**, *11*, 2409084.

[2] C. Pérez-Jiménez, L. Escriche, J. Casabó, *Anal. Chim. Acta* **1998**, *371*, 155-162.

[3] M. B. Saleh, *Talanta* **1998**, *46*, 885-895.

[4] H. Oh, E. M. Choi, H. Jeong, K. C. Nam, S. Jeon, *Talanta* **2000**, *53*, 535-542.

[5] M. R. Ganjali, P. Norouzi, M. Emami, M. Golmohammadi, A. Mouradzadegun, *J. Chin. Chem. Soc.* **2006**, *53*, 1209-1214.

[6] A. Casnati, F. Giunta, F. Sansone, R. Ungaro, M. Montalti, L. Prodi, N. Zaccheroni, *Supramol. Chem.* **2001**, *13*, 419-434.

[7] M. Shamsipur, S. Y. Kazemi, H. Sharghi, K. Niknam, *Fresenius J. Anal. Chem.* **2001**, *371*, 1104-1108.

[8] R. K. Mahajan, M. Kumar, V. Sharma, I. Kaur, *Talanta* **2002**, *58*, 445-450.

[9] M. Arvand-Barmchi, M. F. Mousavi, M. A. Zanjanchi, M. Shamsipur, *Sens. Actuat. B* **2003**, *96*, 560-564.

[10] H. A. M. Arida, R. F. Aglan, S. A. El-Reefy, *Anal. Lett.* **2004**, *37*, 21-33.

[11] S. Peper, C. Gonczy, W. Runde, *Talanta* **2005**, *67*, 713-717.

[12] R. Bereczki, V. Csokai, A. Grün, I. Bitter, K. Tóth, *Anal. Chim. Acta* **2006**, *569*, 42-49.

[13] A. Radu, S. Peper, C. Gonczy, W. Runde, D. Diamond, *Electroanal.* **2006**, *18*, 1379-1388.

[14] H. S. Seo, M. M. Karim, S. H. Lee, *J. Fluoresc.* **2008**, *18*, 853-857.

[15] M. Arvand, M. Moghimi, M. A. Bagherinia, *Anal. Lett.* **2009**, *42*, 393-408.

[16] P. S. Ramanjaneyulu, A. N. Kumar, Y. S. Sayi, K. L. Ramakumar, S. K. Nayak, S. Chattopadhyay, *J. Hazard. Mater.* **2012**, *205*, 81-88.

[17] H. S. Seo, S. H. Lee, *J. Fluoresc.* **2011**, *21*, 747-751.

[18] B. Radaram, T. Mako, M. Levine, *Dalton Trans.* **2013**, *42*, 16276-16278.

[19] M. Shamsipur, H. R. Rajabi, *Microchim. Acta* **2013**, *180*, 243-252.

[20] S. Lin, C. Yang, Z. F. Mao, B. Y. He, Y. T. Wang, C. H. Leung, D. L. Ma, *Biosens. Bioelectron.* **2016**, *77*, 609-612.

[21] S. M. Kang, S. C. Jang, Y. Haldorai, A. T. E. Vilian, M. Rethinasabapathy, C. Roh, Y. K. Han, Y. S. Huh, *RSC Adv.* **2017**, *7*, 48374-48385.

[22] M. Akamatsu, H. Komatsu, A. Matsuda, T. Mori, W. Nakanishi, H. Sakai, J. P. Hill, K. Ariga, *Bull. Chem. Soc. Jpn.* **2017**, *90*, 678-683.

[23] N. Kumar, P. X. Qui, A. Depauw, M. Hemadi, N. T. Ha-Duong, J. P. Lefevre, M. H. Ha-Thi, I. Leray, *New J. Chem.* **2017**, *41*, 7162-7170.

[24] J. Ahn, N. Y. Lim, Y. Choi, M. Y. Choi, J. H. Jung, *Sens. Actuat. B* **2018**, *255*, 325-331.

[25] X. Q. Pham, L. Jonusauskaite, A. Depauw, N. Kumar, J. P. Lefevre, A. Perrier, M. H. Ha Thi, I. Leray, *J. Photoch. Photobio A.* **2018**, *364*, 355-362.

[26] Y. J. Gwon, C. Kim, T. S. Lee, *Sens. Actuat. B* **2019**, *281*, 343-349.

[27] J. Y. Qiu, L. Fu, H. S. Wang, R. F. Zou, Y. J. Zhang, X. Li, A. G. Wu, *New J. Chem.* **2020**, *44*, 2241-2246.

[28] L. Wang, Z. M. Wang, C. Zhou, W. J. Song, C. Y. Sun, *J. Dispersion Sci. Technol.* **2020**, *41*, 1095-1103.

[29] A. Kasprzak, H. Sakurai, *Chem. Commun.* **2021**, *57*, 343-346.

[30] Z. M. Wang, L. Wang, C. Zhou, C. Y. Sun, *RSC Adv.* **2021**, *11*, 10075-10082.

[31] J. Fu, L. Zhang, S. L. Wang, W. L. Yuan, G. H. Zhang, Q. H. Zhu, H. Chen, L. He, G. H. Tao, *J. Hazard. Mater.* **2022**, *425*, e127981.

[32] T. P. Nguy, V. Kilinc, R. Hayakawa, C. Henry-de-Villeneuve, J. M. Raimundo, Y. Wakayama, A. Charrier, *Sens. Actuat. B* **2022**, *351,* e130956.

[33] J. S. Cyniak, L. Kocobolska, N. Bojdecka, A. Gajda-Walczak, A. Kowalczyk, B. Wagner, A. M. Nowicka, H. Sakurai, A. Kasprzak, *Dalton Trans.* **2023**, *52*, 3137-3147.

[34] J. J. Weiss, *Nature* **1969**, *221*, 392-392.

[35] X. Tian, H. Jiang, L. Hu, M. Wang, W. Cui, J. Shi, G. Liu, Y. Yin, Y. Cai, G. Jiang, *Trends Anal. Chem.* **2022**, *157*, 116746.

[36] T. S. Lum, K. Sze Yin Leung, *J. Anal. At. Spectrom.* **2016**, *31*, 1078-1088.

[37] Z. Qu, E. Steinvall, R. Ghorbani, F. M. Schmidt, *Anal. Chem.* **2016**, *88*, 3754-3760.

[38] T. Sikolenko, E. Bou Maroun, L. Noret, M. Legal, T. Karbowiak, *Food Chem.* **2025**, *491*, 145047.

[39] H. Liao, P. K. Dasgupta, *Anal. Chem.* **2016**, *88*, 2198-2204.

[40] X. Li, H. Chang, *Microsyst. Nanoeng.* **2020**, *6*, 66.
